# Genome-based taxonomy and provirus identification in *Halococcus* from hypersaline environments

**DOI:** 10.1016/j.crmicr.2026.100637

**Published:** 2026-06-25

**Authors:** Ruby Setiawan, Jeroen G. Nijland, Sabine Schwarzer, Thomas Hackl, Dian Alfian Nurcahyanto, Ekowati Chasanah, Tessa E.F. Quax

**Affiliations:** a Molecular Microbiology, Groningen Biomolecular Sciences and Biotechnology Institute, University of Groningen, 9747AG, Groningen, The Netherlands; b Eco-evolutionary Bioinformatics, Groningen Institute for Evolutionary Life Sciences, University of Groningen, 9747AG, Groningen, The Netherlands; c Research Center for Biosystematics and Evolution, National Research and Innovation Agency (BRIN), 16911, Cibinong, West Java, Indonesia; d Research Center for Applied Microbiology, National Research and Innovation Agency (BRIN), Cibinong, West Java, 16911, Indonesia

**Keywords:** *Halococcus*, Whole-genome, Provirus, Haloarchaea, Viruses, Archaeal virus

## Abstract

*Halococcus* species are widely distributed in hypersaline environments and possess unusual cell wall structures. However, their genomic diversity, antiviral defenses, and interactions with viruses remain poorly understood. Here, we sequenced and analyzed the genomes of seven *Halococcus* strains isolated from hypersaline habitats. Comparative analyses showed substantial variation in growth dynamics, genome size, and defense repertoires, revealing considerable differences among species within the genus. Growth analyses showed distinct behavior among closely related strains, with slow-growing isolates strongly associated with the presence of proviruses, suggesting a fitness cost of lysogeny. Genome-based metrics, including ANI and dDDH, indicated that five of the seven isolates represent four novel species candidates. Two divergent Caudoviricetes-like proviruses were identified. A CRISPR spacer-protospacer match in one strain suggests virus-host interactions and may indicate maintenance of lysogeny. The low sequence similarity of these proviruses to known archaeal viruses supports the existence of previously unrecognized viral lineages infecting *Halococcus*, providing new insights into the ecology and evolution of this genus in extreme saline environments.

## Highlights

• Halococci differ from other haloarchaea by their thick cell wall structure.
• Currently no viruses are known to infect Halococci.
• Seven novel Halococcus strains were characterized and the genomes sequenced.
• The novel Halococcus strains contain predicted anti-viral defence mechanisms.
• Detected proviruses indicate that Halococci are infected by viruses in nature.

## Graphical abstract

**Figure:**
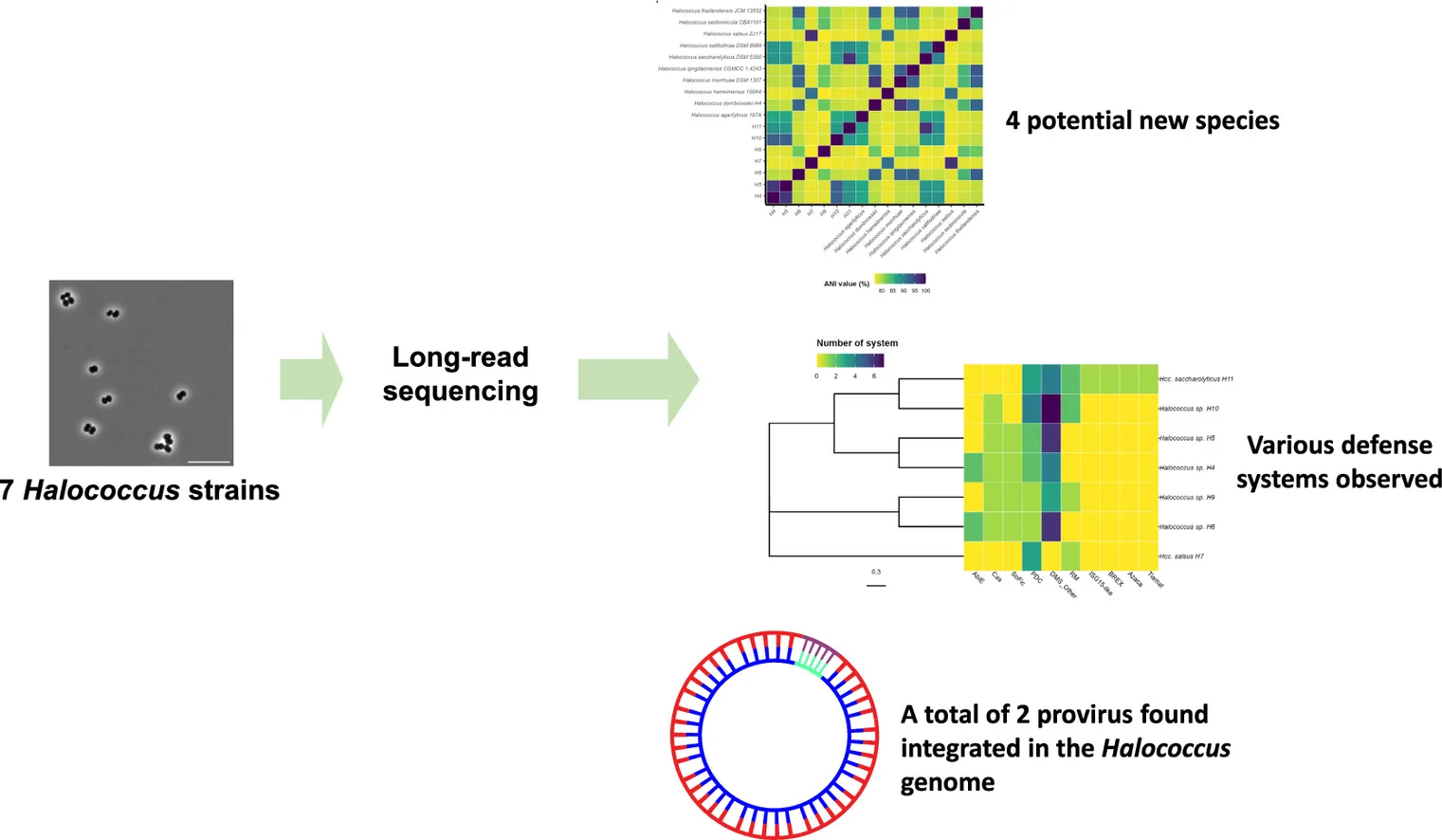

## Introduction

Extremophiles thrive under physicochemical conditions once considered incompatible with life. They serve as valuable models for understanding molecular adaptation to environmental factors and the limits of biological systems (Rothschild, and Mancinelli, 2001). Among the extremophiles, haloarchaea are key representatives of life in hypersaline environments, with a total of 387 species (https://lpsn.dsmz.de) distributed into 84 genera that have been successfully isolated from inland and marine salt environments, as well as from products derived from these environments (such as salt) (Cui, and Dyall-Smith, 2021; Schober et al., 2025) . In order to survive in hypersaline environments, they have evolved mechanisms to withstand high ionic strength, desiccation, fluctuating temperatures, nutritional deficiency, and intense UV radiation (Oren, 2006). This makes haloarchaea a good model for understanding cellular adaptation to extreme dry and saline environments, and as such, they can even serve as a model for life on other planets such as Mars (DasSarma et al., 2020). Like other domains of life, haloarchaea are hosts to viruses. However, the diversity, biology, and evolutionary impact of archaeal viruses are understudied compared with those of viruses infecting bacteria and eukaryotes.

The genus *Halococcus* harbors salt-loving haloarchaea, characterized by coccoid morphology. They are notable for pink to red pigmentation, which is linked to the presence of carotenoids (Grivard et al., 2022; Tichy et al., 2023). The carotenoids also provide UV protection through antioxidant activity. Furthermore, *Halococcus* possesses nucleotide excision repair genes to recover from UV-induced DNA damage (Gudhka, Neilan, and Burns, 2015; Jones, and Baxter, 2017). *Halococcus* cells require at least 15% NaCl for growth and can grow at near-saturated salt concentrations (Chen et al., 2018; Yim et al., 2015). Members of this genus inhabit hypersaline environments such as salt lakes, solar salterns, and evaporitic deposits, ecosystems found worldwide (Chen et al., 2018; Minegishi et al., 2015; Stan-Lotter et al., 2002; Tavoosi et al., 2023; Yim et al., 2014). A remarkable feature that distinguishes this genus from other haloarchaea is its cell envelope composition. Whereas most haloarchaea possess a proteinaceous S-layer, *Halococcus* has a highly sulfated heteropolysaccharide envelope (Albers, and Meyer, 2011; Legat et al., 2013). It was found to consist of glucose, galactose, mannose, N-acetylglucosamine, N-acetylgalactosamine, galactosamine, glucosamine, galacturonic acid, glucoronic acid and a small amount of glycine (Legat et al., 2013; Schleifer, Steber, and Mayer, 1982).This cell envelope forms a rigid cell wall that protects the *Halococcus* from lysis in the absence of salt, a feature uncommon among other haloarchaea (Leuko et al., 2008; Oren, 1994). Although viruses infecting haloarchaea are the most abundant archaeal viruses, isolated today, no free viral particles have been isolated that infect *Halococcus*. It is possible that the unusual surface structure underlies the apparent absence of *Halococcus*-infecting viruses.

Despite increasing genomic data for haloarchaea, the *Halococcus* genomic field remains underdeveloped. Only 19 genomes are currently available in NCBI, including 10 from type strains, often incomplete or of variable quality, reflecting diverse sequencing approaches from short-read assemblies and long-read strategies (Wu, and Ma, 2019). The lack of complete, closed genomes constrains comparative analyses. Although a provirus has been reported in *Halococcus* (Xu et al., 2024), it remains to be determined whether additional proviruses exist but have been overlooked due to incomplete genome assemblies, whether the unique cell envelope influences viral susceptibility, and whether *Halococcus* genomes encode antiviral defense systems indicative of frequent viral encounters. Addressing these questions requires high-quality genomic data capable of revealing integrated viral elements and defense-associated loci such as CRISPR-Cas or restriction-modification systems (Tesson et al., 2022).

In this study, we used long-read genome sequencing from Oxford Nanopore and comprehensive genomic analyses to broaden the genomic representation of the *Halococcus* genus. Our work provides high-quality, complete genomes, identifies putative proviruses and antiviral defense systems, and offers a genomic foundation to explore the interactions between *Halococcus* and potential viruses. As such, it will serve as a stepping stone for future studies of archaeal virus-host dynamics in one of the most extreme microbial niches on earth.

## Materials and methods

### Halococcus strain and growth conditions

Seven uncharacterized *Halococcus* strains were isolated from salt crystals and saline soil samples collected in Indonesia (Supplementary Table S1). The *Halococcus* strains were maintained in 20% MGM liquid consisting of 666 ml 30% artificial salt water (240 g NaCl, 30 g MgCl_2_·6H_2_O, 35 g MgSO_4_·7H_2_O, 7 g KCl, 80 ml of 1 M Tris–HCl (pH 7.2) per liter), 5 g peptone (Oxoid), 1 g Bacto yeast extract (Gibco, Thermo Fisher), and 1 ml of 1 M CaCl_2_ 2H_2_O per liter and addition of 14 g Bacto agar (BD Biosciences) per liter for solid medium (Dyall-Smith, 2009). *Halococcus* spp. were grown in 20% MGM liquid medium at 37 °C with shaking at 150 rpm under aerobic conditions with an initial optical density (OD) of 0.02. Optical density at 600 nm (OD_600_) was measured at regular intervals to monitor growth. Each measurement was taken from three biological replicates, and the average values were plotted to generate growth curves. Furthermore, the maximum growth rates were determined by implementing the smooth.spline model from the grofit package (Kahm et al., 2010) in RStudio. Cell morphology was examined using an Axio Observer 7 inverted microscope (Carl Zeiss Microscopy GmbH, Jena, Germany). A 5 µL aliquot of cell culture was spotted onto a 2% (w/v) agarose pad prepared with 18% salt water to maintain osmotic balance. After the sample droplet had dried, it was gently covered with a coverslip. Observations were performed using a Plan-Apochromat 100 × /1.4 Oil DIC (differential interference contrast) phase contrast objective in PH3 mode. Images were captured using the Zen 3.5 software (Zeiss) under consistent exposure settings for all samples. For each *Halococcus* strain, the diameters of 100 individual cells were manually measured using the straight-line tool in ImageJ Fiji (Schindelin et al., 2012). Measurements were taken along the longest visible axis of each coccus to ensure consistency across clustered cells.

### Genomic DNA extraction

Cells were harvested in the early exponential phase (Leuko et al., 2008). Genomic DNA was extracted using QIAamp DNA Minikit (Qiagen, cat no. 51,304) with a modified protocol. Approximately 3 ml of culture from the early exponential phase was centrifuged at 5000 × g for 5 min at room temperature. The cell pellet was collected and resuspended in 180 µl ELB Buffer (2 mM EDTA, 1.2% (v/v) Triton X-100, 0.02 M Tris–HCl) supplemented with 3.6 µg of lysozyme. The mixture was incubated at 37 °C for 1 h, and 2 µl of RNase (100 mg/ml) was added to the mixture, then vortexed for 15 *sec*. Twenty microliters of Proteinase K (QIAGEN) and 200 µl of AL buffer (QIAGEN) were added to the mixture and incubated at 56 °C for 30 min. Two hundred microliters of 96% ethanol was added to the mixture and mixed thoroughly. The mixture was then transferred to the column, washed, and eluted according to the manufacturer's protocol (QIAGEN).

### Genome sequencing and assembly

The aliquots of extracted gDNA from each strain were used to prepare libraries for whole-genome sequencing on the MinION sequencer. The libraries were prepared using a native barcoding kit v14 SQK-NBD114 from Oxford Nanopore Technologies (ONT, Oxford, United Kingdom) according to the manufacturer's protocol. The sequencing was performed using a MinION R10.4.1 flow cell on an ONT MinION Mk1b sequencing platform, according to the manufacturer’s protocol. Sequencing was controlled using the MinKNOW software v24.11.8, and live basecalling was performed using Dorado (Oxford Nanopore Technologies, 2025) software v1.1.1 in GPU mode with the Super-accurate basecalling v4.3.0, 400 bps model.

The basecalled ONT reads in FASTQ format were filtered to have a minimum length of 1000 bp and performed quality check using SeqKit v2.9.0 (Shen, Sipos, and Zhao, 2024) (Supplementary Table S2), and reads were assembled using Flye v2.9.5-b1801 applying the –nano-hq parameter (Kolmogorov et al., 2019). Medaka v2.0.1 (Oxford Nanopore Technologies, 2024) was used to polish the assembled contigs from Flye. Assembly completeness and potential contamination were verified using CheckM2 v1.1.0 (Chklovski et al., 2023). The obtained assemblies were displayed using pyCirclize (Shimoyama, 2022).

### Taxonomic classification and genome annotation

The phylogenetic position of the *Halococcus* strains and their closest relatives was determined using 16S rRNA and rpoB’ gene-based phylogenetic tree constructed using Neighbor-Joining (NJ) methods (Saitou, and Nei, 1987) in MEGA12 (Kumar et al., 2024). Moreover, the taxonomic position of the *Halococcus* strains was assessed from the assembled genome using TYGS Server (https://tygs.dsmz.de) (Meier-Kolthoff et al., 2022; Meier-Kolthoff, and Göker, 2019). A phylogenomic tree was reconstructed from concatenated single-copy marker genes using the archaeal marker sets from GTDB r226, with the de novo workflow in GTDB-Tk v2.7.2 (Chaumeil et al., 2022). Additionally, digital DNA-DNA hybridization (dDDH) and orthologous average nucleotide identity (orthoANI) values were determined using the Genome-to-Genome Distance Calculator (GGDC) (https://ggdc.dsmz.de) web servers (Meier-Kolthoff et al., 2022) and PyOrthoANI (Larralde, Zeller, and Carroll, 2025; Lee et al., 2016) to assess the genetic similarity between strains. Open Reading Frames (ORFs) were predicted using Prokka v1.14.6 (Seemann, 2014) with the genus parameter set as *Halococcus*. Furthermore, the ORFs were annotated with BlastKOALA (Kanehisa, Sato, and Morishima, 2016) and eggNOG-mapper v2 (Cantalapiedra et al., 2021) to identify genes with KEGG annotations and COG category, respectively. Comparative metabolic analysis from the annotated KEGG genes was performed using KEGG Mapper Reconstruct v5 (Kanehisa, Sato, and Kawashima, 2022).

### Identification of the defense system and provirus prediction

Viral defense systems were identified using the Prokaryotic Antiviral Defence Locator (PADLOC) v2.0.0 (Payne et al., 2021) and CRISPR-CasFinder web tool (https://crisprcas.i2bc.paris-saclay.fr/CrisprCasFinder/Index) (Couvin et al., 2018) from the assembled genomes to identify defense mechanisms against viral infection, including CRISPRCas, restriction-modification, and abortive infection. The obtained CRISPR array was then searched for similarity using BLAST against the spacers sequences inthe CRISPRCas++ database (Pourcel et al., 2019). Moreover, to check the probability of provirus and potential plasmid in the assembled genomes, we utilize geNomad v1.9.0 (Camargo et al., 2024) analysis from the genome sequences. The obtained provirus region was then annotated using Pharokka (Bouras et al., 2023) and Phold (Bouras et al., 2026), and viral relationships were further assessed using ViPTree (Nishimura et al., 2017) based on all-versus-all tBLASTx comparison of viral genomes. The obtained Cas genes or provirus were aligned using Mmseqs2 (–easy-search and –search-type 4) (Steinegger, and Söding, 2017), and the results were visualized using the gggenomes package (Hackl et al., 2024) in RStudio. Potential plasmids were then compared against the plasmid database using pLAST with Mmseqs2 model to identify related plasmids (Krakowski et al., 2025).

#### Provirus induction

*Halococcus* strains predicted to harbor proviruses were cultured as described in the *“Halococcus strain and growth condition”* section until reaching OD_600_ of approximately 0.5–1.0, at which point mitomycin C (Serva, cat.no. 29,805.01) was added to a final concentration of 2 or 5 µg/ml. Parallel cultures without mitomycin C served as untreated controls. Cultures were incubated at 37 °C with shaking at 50 rpm, and the OD_600_ was monitored over 96 h. At the end of incubation, culture supernatants were collected, boiled, and used as templates for qPCR amplification of viral fragments. Primes used for qPCR are listed in Supplementary Table S3. Each qPCR was carried out in 12.5 µl containing 1x SensiFAST SYBR HI-ROX Mix, 0.4 µM of each primer, and 2 µl of each template. Amplification was performed on Bio-Rad CFX Connect Real-Time System (Bio-Rad Laboratories) with an initial denaturation at 95 °C for 3 min, followed by 40 cycles of 20 s denaturation at 95 °C, 30 s annealing at the designated annealing temperature and 30 s elongation at 72 °C. Product specificity was verified by melting curve analysis from 65 °C to 95 °C with 0.5 °C increments. Reactions were performed in technical triplicates, with nuclease-free water and genomic DNA included as non-template controls and positive controls, respectively.

#### Electron microscopy

*Halococcus* sp. H4 was grown in 20% MGM liquid at 37 °C until reaching OD_600_ ∼1.5. The cells were collected by centrifugation at 4000x g for 15 min and transferred to a gold copper type A carrier (Leica) and covered with an aluminium type B carrier (Leica). Cells were fixed with high-pressure freezing using the Leica EM ICE and subsequently freeze-substituted in freeze-substitution medium (1% (w/v) OsO_4_, 0.5% uranyl acetate in acetone with 5% water) using quick freeze-substitution method (McDonald, and Webb, 2011) in a Leica automated freeze-substitution machine. Following embedding in Epon resin, ultra-thin section (∼100 nm) were prepared and collected on formvar/carbon-supported copper grids and inspected using CM120 (Phillips) transmission electron microscope.

## Results

### Strains display typical Halococcus growth and morphology

**Fig. 1:**
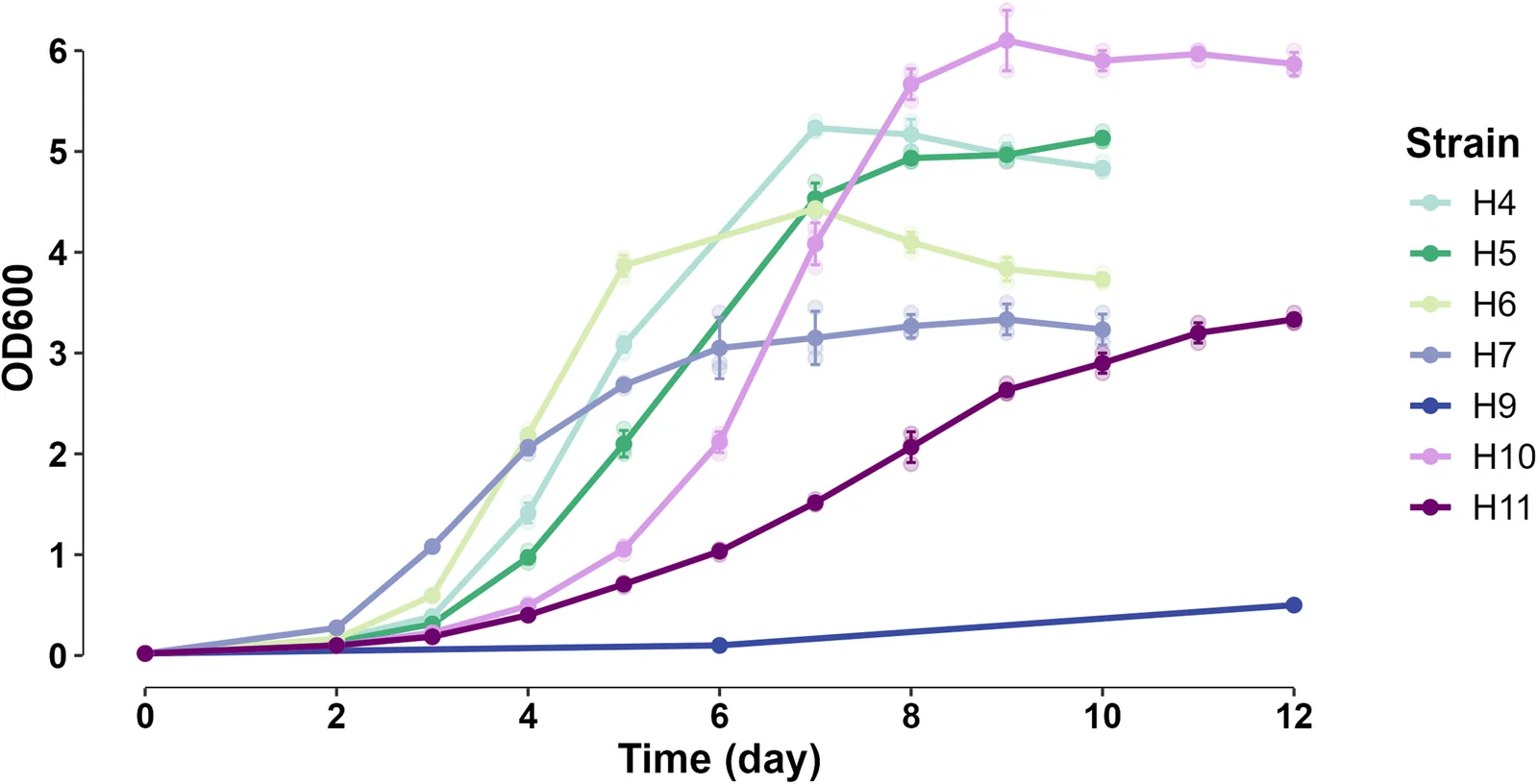
Comparative growth dynamics of *Halococcus* strains in 20% MGM medium at 37 °C were observed using optical density measurements at 600 nm over 10 days. Points represent the average values, and the error bars represent the standard deviation from three biological replicates.

**Fig. 2:**
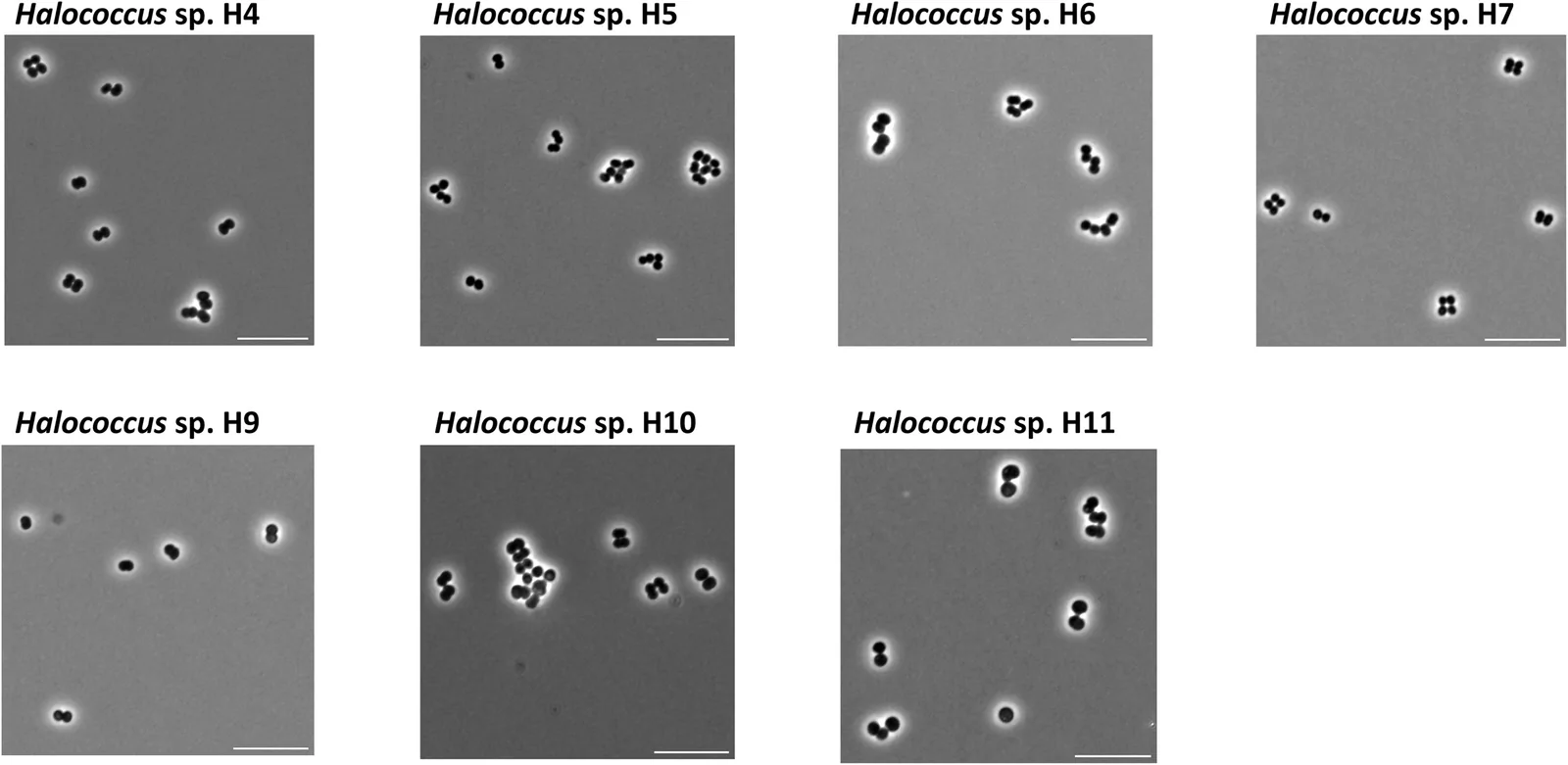
Cell morphology of *Halococcus* strains. Cells of each strain were observed at mid-exponential phase using phase contrast microscopy. All strains exhibited a similar coccoid morphology, with cells appearing predominantly in pairs and occasionally forming tetrads or irregular clusters of paired cells. Scale bars: 10 µm.

In this study, seven uncharacterized *Halococcus* strains (named H4, H5, H6, H7, H9, H10 and H11) were isolated from salt crystals and saline soil from samples collected in Indonesia (Supplementary Table S1). All *Halococcus* strains grew well in 20% liquid MGM, although showing different maximal growth rates (Fig. 1). Strains H4, H5, H6, and H7 reached the stationary phase after 6 - 8 days of incubation, whereas strains H10 and H11 reached maximum OD after 9 - 12 days of incubation. Strain H9 showed no growth peak after 12 days, reaching an OD of only 0.5. Further incubation of strain H9 up to 19 days shows an increase in OD to 0.9 (Supplementary Fig. S1). A longer incubation of the H9 culture induces salt crystal precipitation. The high final OD values (OD600 > 1.0) are commonly observed in haloarchaea and have been reported previously in *Haloferax gibonsii* and *Haloarcula californiae,* which can reach OD around 2.5 and 4.0, respectively (Schwarzer et al., 2021; Tittes et al., 2024). The calculated maximum growth rates (µ_max_) of the isolated strains ranged from 0.245 to 1.26 days^–1^ (Supplementary Table S4). Therefore, we grouped these *Halococcus* strains into three categories: fast-growing (H4, H5, H6, H7), where µ_max_ ≥0.9 days^–1^, moderate-growing (H10, H11), where 0.5 ≤ µ_max_ ≤0.9 days^–1^, and slow-growing (H9) where µ_max_ < 0.5 days^–1^. Comparative metabolic analysis of KEGG annotated genes, focusing on carbon central metabolism and nutrient acquisition showed that growth variation was related to the difference in central carbon metabolic capacity rather than to nutrient acquisition according to genomic prediction (Supplementary Fig. S2). The morphology of the *Halococcus* strains was analysed during the mid-exponential phase using phase-contrast microscopy, which showed that all seven strains exhibit a similar morphology. Cells appeared as cocci, mostly in pairs, with some forming tetrads or irregular clusters of paired cells (Fig. 2). Manual measurements of 100 cells per strain revealed an average cell diameter of 1.43 ± 0.18 µm for H4, 1.25 ± 0.18 µm for H5, 1.55 ± 0.27 µm for H6, 1.21 ± 0.17 µm for H7, 1.43 ± 0.23 µm for H9, 1.37 ± 0.20 µm for H10, and 1.73 ± 0.38 µm for H11, with no significant differences observed between strains. Thin section of *Halococcus* sp. H4 cells revealed a thick envelope with an irregular outer layer (Supplementary Fig. 3), consistent with previously described *Halococcus* species (Denner et al., 1994; Goh et al., 2006; Wang et al., 2007).

### Genome sequencing reveals the presence of several potential novel species

The genomes of the *Halococcus* strains were sequenced on the MinION sequencer with Native Barcoding Kit v14. De novo genome assembly was performed using Flye. The assembled genome sizes of these *Halococcus* strains ranged from 3.3 to 4.0 M base pairs, and the GC content ranged from 61.68 to 64.42% (Table 1, Supplementary Fig. S4). We obtained a circular chromosomal genome for strains H4, H5, H6, H7, H9, and H10, comprising 2952,034 bp, 3210,452 bp, 2928,746 bp, 3106,595 bp, 2870,346 bp, and 3407,509 bp, respectively. For strain H11, the chromosomal genome was fragmented into several contigs, which were associated with mobile genetic elements regions. Along with the main chromosome, we also identified potential plasmids in the different strains, which are listed in Supplementary Table S5.

**Table 1:** Genomic features of *Halococcus* strains.

	*Halococcus* H4	*Halococcus* H5	*Halococcus* H6	*Halococcus* H7	*Halococcus* H9	*Halococcus* H10	*Halococcus* H11
Total length (bp)	3,558,933	3,514,208	3,338,529	3,911,851	3,733,640	3,873,436	4,074,520
Largest contig (bp)	2,952,034	3,210,452	2,928,746	3,106,595	2,870,346	3,407,509	1,800,605
Contigs	6	2	5	3	9	4	24
GC content (%)	64.38	64.42	63.84	63.75	61.68	63.77	63.01
CDS	3,676	3,572	3,411	3,917	3,912	3,944	4,119
CDS with KEGG number	1,432	1,426	1,385	1,543	1,457	1,513	1,561
CDS with COG category	2,696	2,631	2,657	2,899	2,794	2,894	3,044
RNA genes							
rRNA	3	3	7	4	3	3	3
tRNA	48	48	47	47	48	47	54

The sequences of the 16S rRNA gene and rpoB’ gene were obtained from the draft genomes and used as queries to search for related sequences in the GenBank database. All seven *Halococcus* isolates showed high 16S rRNA gene similarity (≥ 99%) to known species, with the closest match summarized in Supplementary Table S6. The rpoB’ gene sequence provides lower identity values as compared to the 16S rRNA gene (Supplementary Table S7), and comparison of 16S RNA and rpoB’ BLASTn results revealed both consistent and conflicting matches among strains. Of the seven isolates, only three isolates (H7, H10, H11) had a consistent closest match between the 16S rRNA gene and rpoB’ gene. These differences underscore the value of using multiple phylogenetic markers for more accurate taxonomic resolution in *Halococcus* (Cui et al., 2024).

**Fig. 3:**
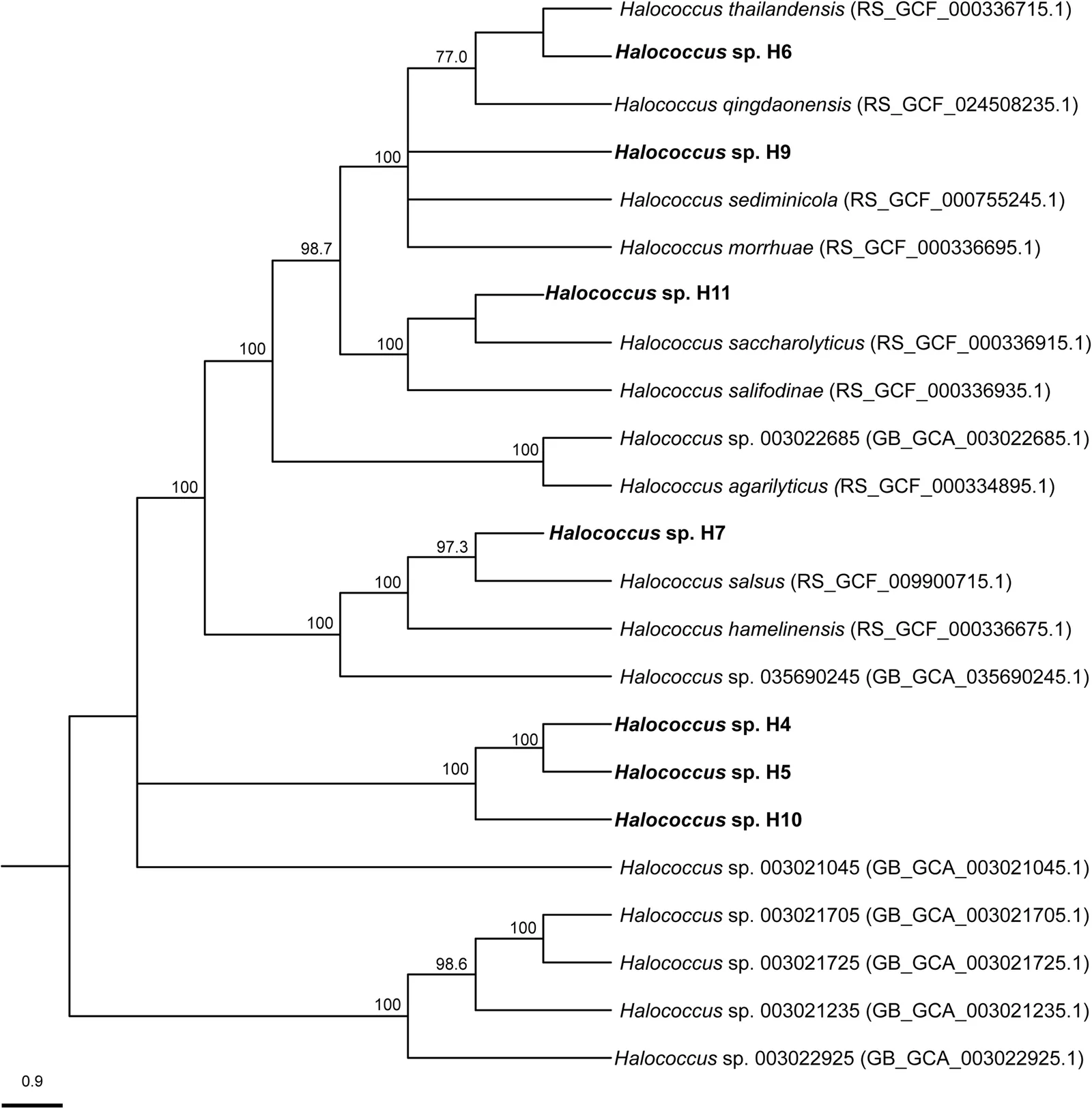
Phylogenomic tree showing the relationship among *Halococcus* strains analyzed in this study (in bold) and their related strains based on concatenated single copy gene markers. Bootstrap values >70% are shown at branch points (based on 1000 replicates). Sequence accession numbers are shown in parentheses.

The neighbor-joining phylogenetic tree of the 16S rRNA gene and rpoB’ gene (Supplementary Fig. S5) revealed that the isolated strains clustered into three groups within the *Halococcus* clade, with *Halalkalicoccus tibetensis* as the outgroup. Both phylogenetic trees show that strains H7 and H11 are closely related to *Halococcus salsus* and *Halococcus saccharolyticus*, respectively, with bootstrap values≥90%. Strains H4, H5, and H10 were grouped into a new clade and form a sister clade with *Halococcus agarilyticus, Halococcus saccharolyticus*, and *Halococcus* sp. H11 in the 16S phylogenetic tree. Meanwhile, in rpoB’ tree, strains H4, H5, H11 and *Halococcus saccharolyticus* form a sister clade with strain H10. This incongruence between the 16S rRNA and rpoB’ phylogenies indicates that single-gene markers provide conflicting resolutions for the relationship among these strains. To resolve these conflicts, a phylogenomic tree was reconstructed from a concatenated single-gene marker (Fig. 3). The phylogenomic tree shows that strains H4, H5, and H10 form a distinct clade, clearly separated from the *Halococcus* type species, indicating that these strains are evolutionarily divergent from the currently described taxa. This placement suggests they may represent a novel lineage within the genus. Meanwhile, strains H6, H7, H9, and H11 were closely related to *Halococcus thailandensis, Halococcus salsus, Halococcus sediminicola,* and *Halococcus saccharolyticus,* respectively.

**Fig. 4:**
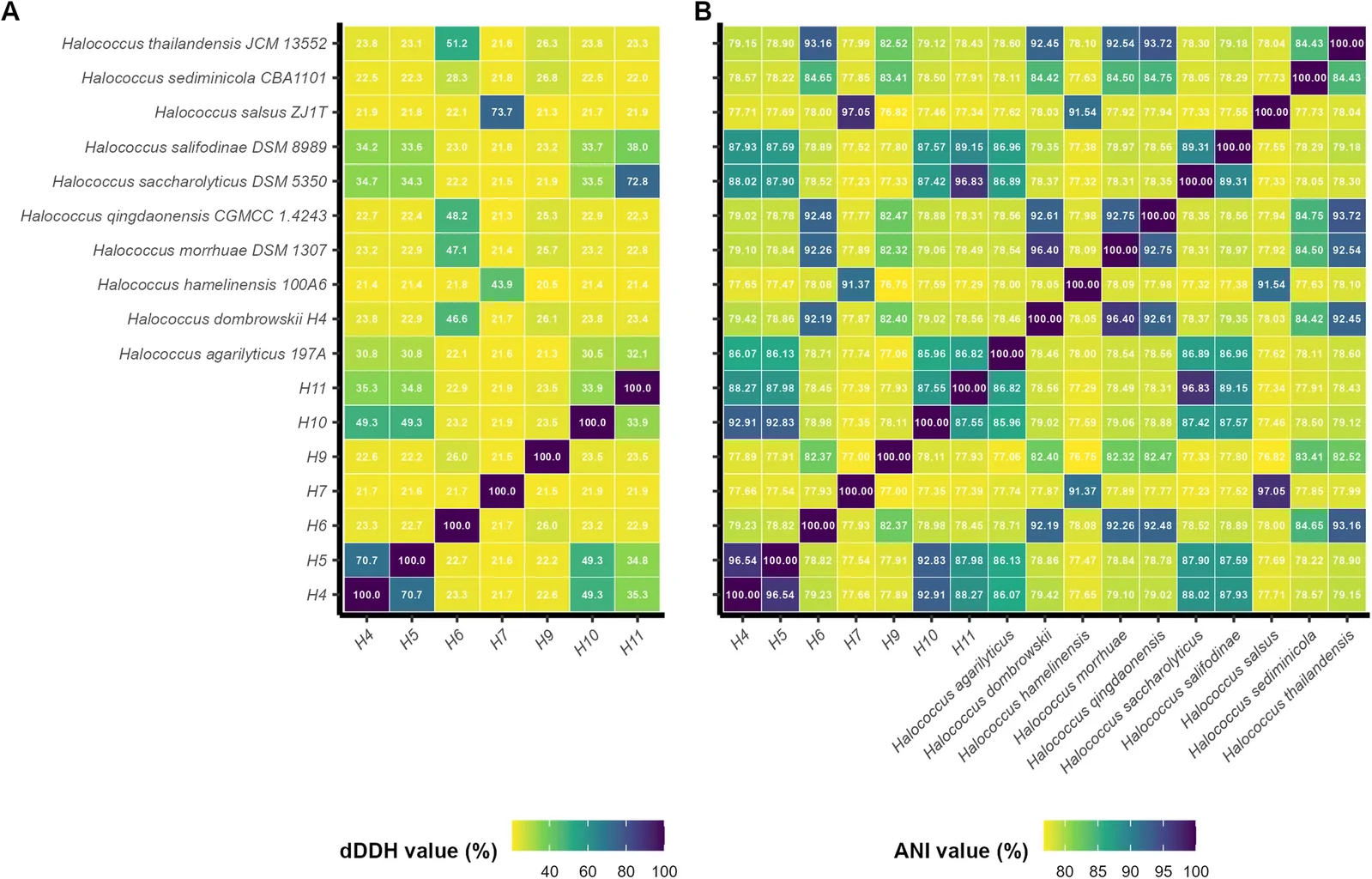
Classification of sequenced *Halococcus* genomes (A) Pairwise comparison of the digital DNA-DNA hybridization (dDDH) using GGDC (https://ggdc.dsmz.de) (Meier-Kolthoff et al., 2013a, 2022) (B) Orthologous average nucleotide identity (orthoANI), calculated using PyOrthoANI (Larralde, Zeller, and Carroll, 2025; Lee et al., 2016). Values between isolated strains and the type strains of members of the *Halococcus* genus are displayed as colors ranging from light green (low) to blue (high), as shown in the key.

Next, we conducted pairwise genome comparisons, including dDDH and orthoANI, to confirm the species boundaries of the isolated strains using the recommended threshold values, namely 70% (Meier-Kolthoff et al., 2013a, 2013b) and 95–96% (Chun et al., 2018; Richter, and Rosselló-Móra, 2009), respectively. Five of the seven isolated strains had dDDH and orthoANI values below the species boundary cut-off values against the available *Halococcus* type strain genomes. Strains H4 and H5 shared a 70.8% and 96.5% dDDH and ANI value, respectively, suggesting that these strains belong to the same species (Fig. 4). Moreover, strain H7 and *Hcc. salsus* shared 77.4% dDDH and 97.0% ANI, while strain H11 shared 73.6% dDDH and 96.8% ANI with *Hcc. saccharolyticus*. These results suggest that strain H7 is a species of *Hcc. salsus,* and H11 is a species of *Hcc. saccharolyticus*. H4, H5, H6, H9, and H10 represent novel species in the genus *Halococcus,* with strains H4 and H5 identified as the same species.

### Genome analysis indicates the presence of proviruses

**Fig. 5:**
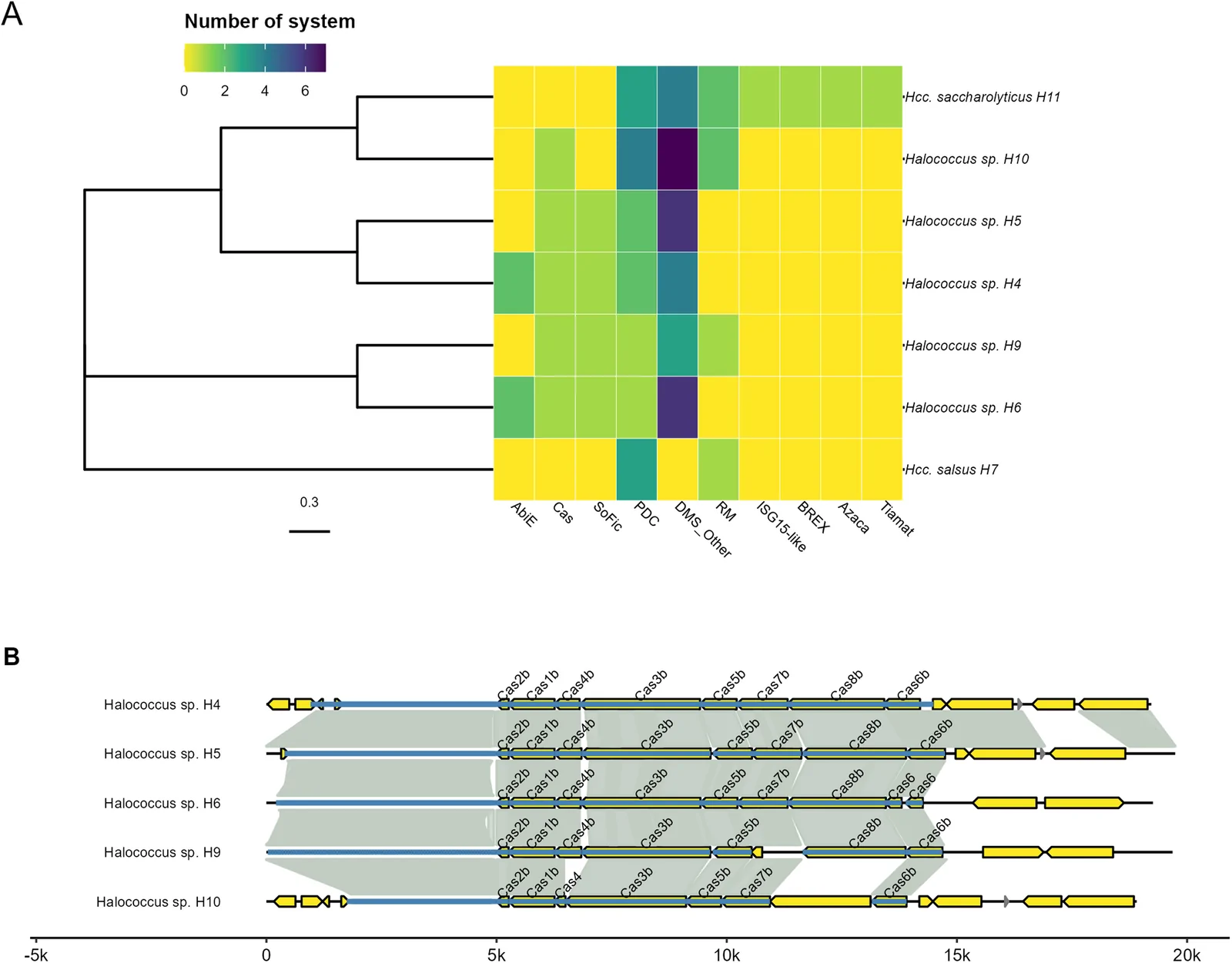
Defense systems in the *Halococcus* strain genomes. (A) Distribution of different defense systems in the *Halococcus* strain genome, based on the presence or absence of genes associated with each defense system as predicted by PADLOC (Payne et al., 2021). Clustering was performed using presence or absence data only and did not account for gene copy number. The tree is constructed from the 16S gene, and the color represents the number of systems detected in each genome as shown in the key. (B) Synteny graph of the CRISPR-Cas system detected in *Halococcus* strains by CRISPR-CasFinder (Couvin et al., 2018), classified as Cas type I-B.

In order to explore the presence of halococci viruses, the distribution of defense systems against foreign genetic elements in the *Halococcus* strains was analyzed. This revealed a diverse range of anti-viral defense systems, with notable variation in both the number and types of systems present (Fig. 5A). Phage Defense Candidate (PDC) and other DNA modification systems (DMS_other) were the most prevalent in the new *Halococcus* genomes, and they were detected in all strains, with DMS_other reaching up to seven variants in strain H10 but not detected in strain H7. In contrast, systems such as ISG15-like, BREX, Azaca, and Tiamat were rare, detected only in strain H11.

The CRISPR-Cas system was found in five of seven strains, indicating widespread adoption among the isolated strains. SoFic, restriction-modification (RM), and abortive infection (AbiE) were variably distributed (Fig. 5A). Among the strains that possess RM systems, two types of RM systems were detected: type I RM system in strains H7 and H11 and the type II RM system in strains H9, H10, and H11. Interestingly, the subtype of type II RM, notably type IIG, is also found in strain H10 (Fig. 5A). Although type II RM systems have been reported in Halobacteriales, including the type IIG, they appear to be less common in *Halococcus* than type I RM (Payne et al., 2021). Abortive infection systems appeared only in strains H4 and H6. Although the strains H4 and H5 are of the same species, they have different defense systems which is due to the presence of plasmids containing the defense systems in strain H4. Notably, strain H7 lacked several major systems, indicating possible reduced defense complexity or the presence of alternative defense mechanisms.

Overall, the distribution of defense systems highlights both shared and unique antiviral strategies among the *Halococcus* strains. This pattern suggests differential evolutionary pressures and possible strain-specific adaptation to viral threats.

To investigate the CRISPR-Cas loci present in the five *Halococcus* strains, CRISPR-CasFinder was used, revealing that all detected CRISPR-Cas systems found in these strains belong to Cas type I-B (Fig. 5). In strain H6, the Cas6b gene was predicted to encode two Cas6 genes by PADLOC. These two Cas6 genes show conserved synteny with other Cas6b, suggesting they are functionally maintained and not pseudogenes. Moreover, strain H9 lacked the Cas7b gene, while the synteny of this gene shows high similarity to that of other Cas7b genes. On the other hand, strain H10 was missing the Cas8b gene. Based on the synteny graph, no similarity was found between these genes and their respective homologs. A BLAST search of the CRISPR spacers against the CRISPR repeat database showed no hits to known viruses in the database (Supplementary Table S8).

**Fig. 6:**
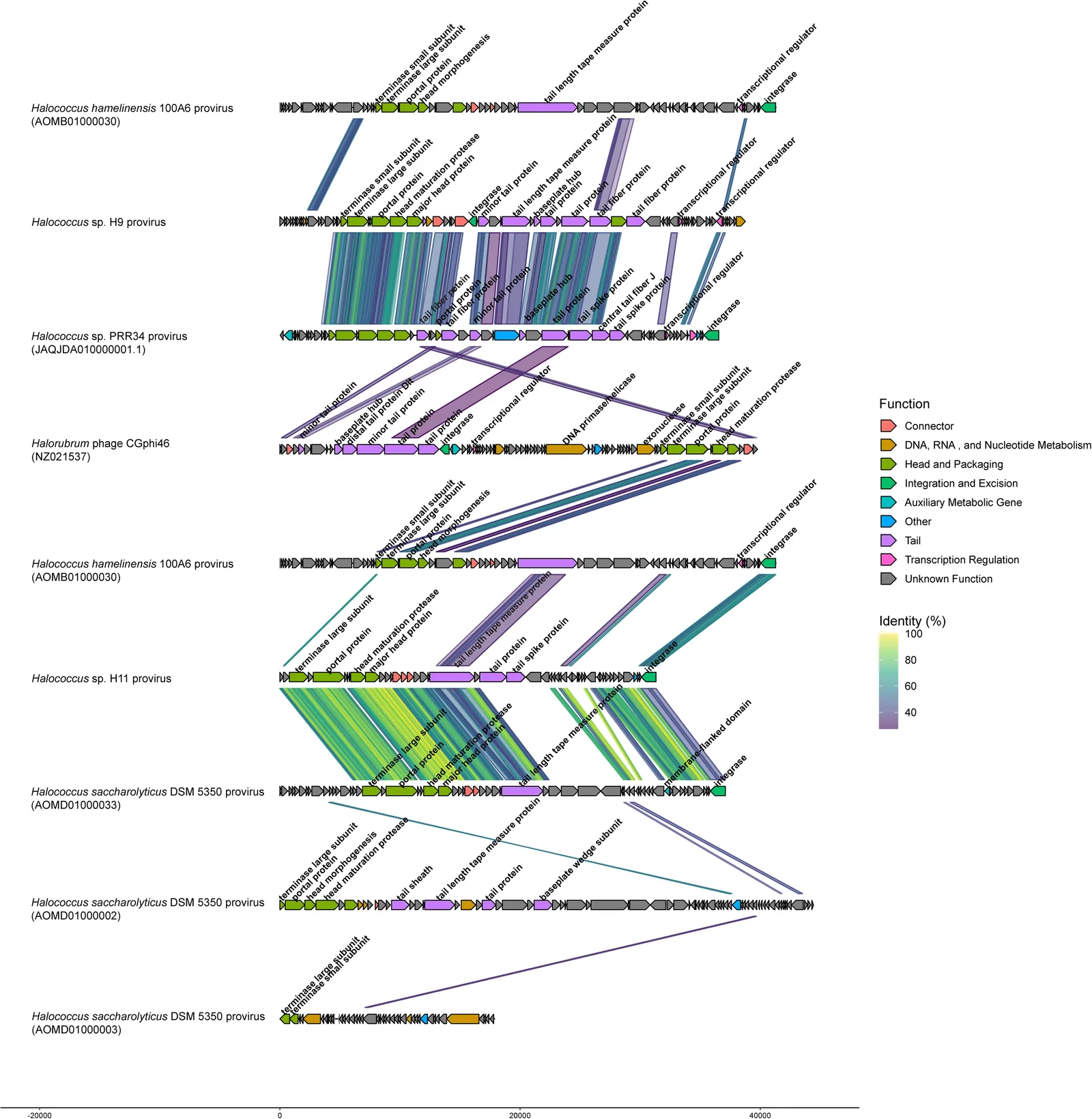
Synteny graph of the *Halococcus* sp. H11, *Hcc. hamelinensis* provirus (AOMB01000030), *Hcc. saccharolyticus* DSM5350 (AOMD01000033), *Halococcus* sp. H9 provirus, *Halococcus* sp. PRR34 provirus (NZ_JAQJDA010000001.1), and *Halorubrum* phage CGphi46 (NC_021537). The proviruses were predicted using geNomad, except the provirus from *Hcc. hamelinensis* 100A6, which was adapted from (Xu et al., 2024). Arrows represent the ORFs, and color indicates functional categories of the annotated proteins, as shown in the legend. Colored block between the genomes denotes regions of DNA sequence homology as determined by MMseqs2 (Steinegger, and Söding, 2017). Sequence accession numbers are shown in parentheses.

**Fig. 7:**
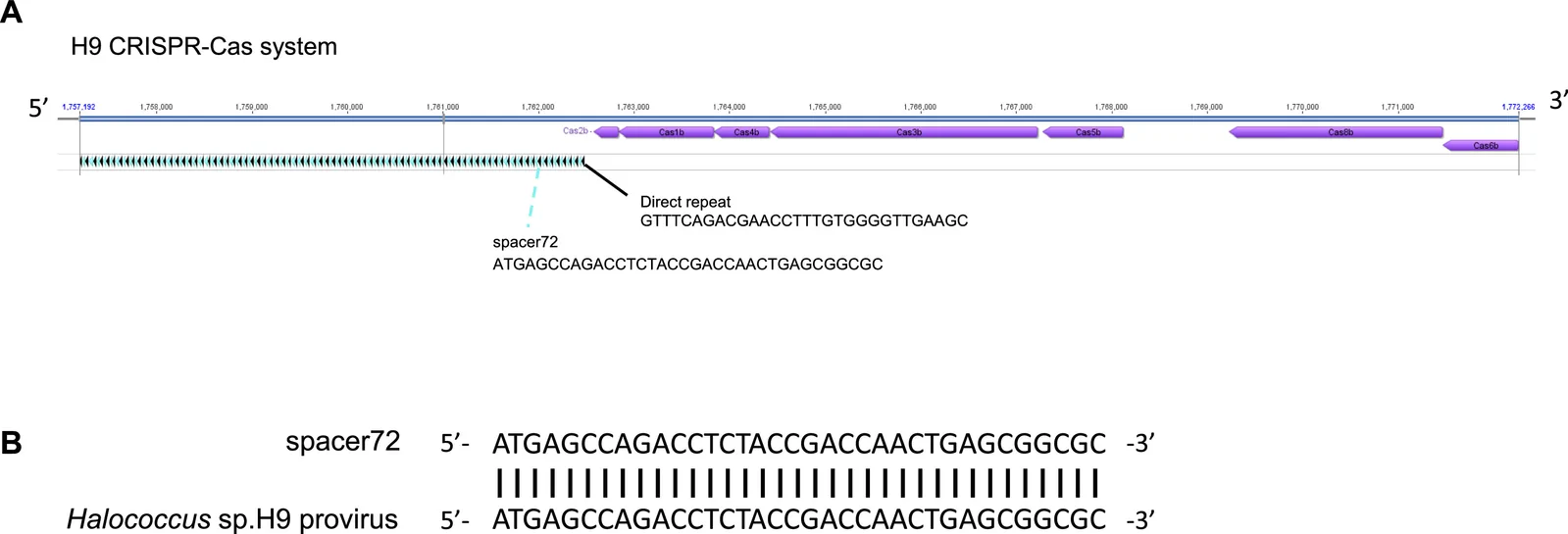
H9 CRISPR-Cas system. (A) Schematic representation of the type I-B CRISPR-Cas system in the *Halococcus* sp. H9. The purple arrows indicate the Cas proteins, the short black arrows and light blue arrows represent the direct repeats and spacers (B). Spacer and protospacer match between the CRISPR system (spacer72) and the H9 provirus.

We also investigated the presence of proviruses in the assembled genomes using geNomad with end-to-end parameters against the genomad database. The predicted provirus sequences were subsequently annotated using Pharokka, followed by Phold. Two out of seven *Halococcus* genomes harbor a provirus, H9 and H11, with lengths of 38,772 bp and 31,324 bp, respectively (Table 3). The predicted *Halococcus* proviruses were compared at both nucleotide and protein levels against the known archaeal tailed virus genomes and previously reported *Halococcus* provirus (Xu et al., 2024) using MMseqs2. In this initial comparison, only low similarity was detected, including provirus in *Halococcus hamelinensis* 100A6 and *Halorubrum* phage CGphi46 (Fig. 6). The predicted proviruses were classified as Caudoviricetes, which was also supported by the presence of the major head protein and tail protein in the annotated provirus sequence. To further assess their relatedness within the genus, we next screened the publicly available *Halococcus* genomes from the NCBI database for additional proviruses and identified four candidates from two *Halococcus* strains (*Halococcus* sp. PRR34 and *Hcc*. saccharolyticus DSM 5350). Compared with these newly identified *Halococcus* proviruses, our sequence showed considerably higher similarity (Fig. 6). The provirus from *Halococcus* sp. H9 shares 27.4% similarity with provirus in *Halococcus* sp. PRR34, and provirus in *Halococcus* sp. H11 shares 50.9% similarity with provirus in *Hcc. saccharolyticus* DSM 5350. Further analysis of the provirus against the spacers revealed that spacer_72 from strain H9 CRISPR system matches with the provirus in strain H9 with 100% nucleotide identity over 100% query coverage of 35 bp (Fig. 7, Supplementary Table S8), specifically in an ORF annotated as a hypothetical protein. Although protospacer adjacent motif (PAM) recognition has been characterized in some haloarchaea type I-B systems, such as *Haloarcula hispanica* and *Haloferax volcanii* (Li, Wang, and Xiang, 2014; Maier et al., 2013), PAM usage is not universal and may vary among species. Therefore, a PAM could not be confidently assigned for the *Halococcus* protospacer match based on a single target sequence alone. This suggests that the CRISPR system is probably active against the provirus, possibly contributing to the maintenance of a lysogenic state. Cells were treated with mitomycin C in order to test if the proviruses could be induced to enter the lytic cycle. This treatment showed no decrease in the cell density compared to the untreated control (Supplementary Fig. S6), indicating the absence of progressive cell lysis. qPCR analysis of the culture supernatant showed no increase in the viral fragment prior to the induction with mitomycin C after 96 h (Supplementary Fig. S7). Together, these results indicate that the predicted proviruses were not inducible under the conditions tested. It cannot be ruled out that they would be induced under other environmental conditions.

**Table 3:** Predicted proviruses from *Halococcus* genomes.

Isolate	Contig name	Position provirus	Length (bp)	Number of genes	Virus score	Number of viral hallmark genes	Taxonomy	Closest relatives (% similarity)
*Halococcus* sp. H9	h9_1	1,490,070-1,528,841	38,772	63	0.8308	3	Caudoviricetes	*Halococcus* sp. PRR34 provirus (27%)
*Halococcus* sp. H11	h11_1	1,210,751-1,248,374	31,324	42	0.8746	4	Caudoviricetes	*Halococcus saccharolyticus* DSM 5350 provirus(50.9%)
*Halococcus* sp. PRR34 (JAQJDA000000000.1)	JAQJDA010000001.1	580,742-617,284	36,543	48	0.8913	3	Caudoviricetes	
*Halococcus saccharolyticus* DSM 5350 (GCA_000336915.1)	AOMD01000033.1	12,416-49,511	37,096	55	0.9181	3	Caudoviricetes	
*Halococcus saccharolyticus* DSM 5350 (GCA_000336915.1)	AOMD01000002.1	95,856-153,638	57,783	97	0.9027	6	Caudoviricetes	
*Halococcus saccharolyticus* DSM 5350 (GCA_000336915.1)	AOMD01000003.1	1–17,913	17,913	38	0.8827	2	Caudoviricetes

## Discussion

We successfully sequenced and analyzed genomes of seven novel *Halococcus* strains. The results reveal notable variation in growth behavior, genome composition, and defense systems, offering new insights into the adaptive strategies of *Halococcus* in hypersaline environments. We also provide high-quality draft genomes of the *Halococcus* strains, thereby increasing the number of *Halococcus* genomes available in public databases.

Growth curve analysis demonstrated that the isolates exhibited distinct growth dynamics, indicating physiological variability even among closely related strains. Differences in lag-phase duration and maximum cell density suggest adaptation to specific microenvironments or differential nutrient utilization capacities. Phase contrast microscopy revealed a consistent coccoid morphology across all isolates, confirming that the observed growth variation was not associated with detectable morphological differences. Two of the three slow-growing strains, H9 and H11, contained proviruses, indicating that these negatively impacted their growth. This physiological phenomenon was confirmed in bacteria, where prophages negatively affected doubling time (Liu, Hu, and Niu, 2023) and deletion of the provirus from haloarchaeal genomes improved growth rates of the hosts (Di Cianni et al., 2025; Turgeman-Grott et al., 2025). However, this association in *Halococcus* is correlative and should not be interpreted as evidence of a direct causal effect. Further experimental validation will be required to determine whether provirus carriage directly affects *Halococcus* growth. Other genomic differences among strains, such as variation in metabolic potential or additional regulatory factors, may also contribute to the observed growth differences (Sun et al., 2020).

At the genomic level, the isolates displayed variable genome sizes and numbers of contigs. The GC content and coding density largely conformed to the previously described *Halococcus* genome, ranging from 60 to 66% (Chen et al., 2018; Denner et al., 1994; Goh et al., 2006; Minegishi et al., 2015; Namwong et al., 2007; Stan-Lotter et al., 2002; Wang et al., 2007; Yim et al., 2014), yet the number of predicted coding sequences and functional categories (COG and KEGG) varied across isolates (Supplementary Table S9). These differences may indicate lineage-specific gene acquisition or loss, particularly in genes related to transport and metabolism, which are critical for survival in extreme salinity .

Phylogenetic analyses of the 16S rRNA and rpoB’ genes placed the isolates within the *Halococcus* genus. Both phylogenetic trees consistently clustered the *Halococcus* isolates into three major lineages, highlighting substantial diversity. However, a different topology for Group II was observed in both trees. In the rpoB’ phylogenetic tree, Groups II and III formed a sister clade rather than Group II associated with Group I as in the 16S rRNA tree (Fig. 3). This discrepancy likely reflects differences in evolutionary constraints and substitution patterns between the rpoB’ and 16S rRNA genes, as well as differences in the algorithm used for phylogenetic tree reconstruction (Minegishi et al., 2010; Yim et al., 2014). Despite these differences, both markers placed the strains in their respective reference groups, suggesting that some strains may be candidates for a new species. These results demonstrated the advantages of using rpoB’ as a complement marker for the 16S rRNA in *Halococcus* (Cui et al., 2024). For these isolated *Halococcus* strains, the recommended threshold for species demarcation based on the 16S rRNA genes was not applicable because the gene is highly conserved among species within the genus and not reliable for species level discrimination (Cui et al., 2024; Minegishi et al., 2015). Instead, the Overall Genome Relatedness Index (OGRI) value including dDDH, ANI, Average Amino Acid Identity (AAI), and the Percentage of Conserved Protein (POCP), was more accurate for determining novel species and resolving taxonomic issues in Haloarchaea (Barco et al., 2020; De La Haba et al., 2021). The pairwise genome comparison values of the *Halococcus* isolates indicated that five out of seven isolated strains, with dDDH and ANI values below the species demarcation threshold, represent four novel species candidates (Fig. 4).

The defense repertoires of the *Halococcus* strains show a broad but uneven landscape, with CRISPR-Cas and several DNA modification systems dominating the antiviral arsenal. The CRISPR-Cas type I-B system, detected in five strains, highlights its broad distribution and aligns with a previous report indicating that type I systems are widespread in Archaea except in Thermoproteota (Maier et al., 2017; Makarova et al., 2025). Meanwhile, the prevalence of PDC and DMS_other across nearly all strains, with an exceptional expansion of DMS_other in strain H10. The PDCs are distributed across archaea, and this defense system remains an uncharacterized defense strategy in haloarchaea (Martínez-Alvarez, and Peng, 2026). The genes identified in DMS_other show similarity with genes in the methylation-associated defense system (MADS), Shango, DNA phosphorothioate modification, and BREX (Goldfarb et al., 2015; Maestri et al., 2024; Millman et al., 2022; Xiong et al., 2019) (Supplementary Table S10). Therefore, DMS-other should not be interpreted as a distinct functional system, but rather as a heterogeneous collection of DNA modification and defense system genes and require further investigation to determine their functions (Payne et al., 2021). In contrast, the rarity of ISG15-like, BREX, Azaca, and Tiamat in the analyzed *Halococcus* genomes, suggests either sporadic acquisition or a limited selective advantage under the environmental conditions shaping these *Halococcus* populations. The low number of Azaca and Tiamat found in *Halococcus* corresponded to the distribution of these genes in the RefSeq database, which included only 2 and 20 genomes out of 382 archaeal complete genomes, respectively (Millman et al., 2022). Meanwhile, the BREX system is abundant in archaea, but not evenly distributed, and rare in *Halococcus*, indicating that this system undergoes an extensive horizontal transfer across microbial species (Goldfarb et al., 2015). These differences imply that *Halococcus* strains experience distinct ecological pressures and rely on diverse, strain specific strategies to counter mobile genetic elements, including plasmids and viruses (Dyall-Smith, and Pfeiffer, 2024; Xu et al., 2024).

Two proviruses were predicted in two different strains. The predicted proviruses were head-tailed viruses belonging to the class Caudoviricetes. Furthermore, analysis of spacers from strain H9 revealed a spacer-protospacer match with its provirus. This might imply that the spacer was acquired through a viral infection (Li et al., 2014), and that this spacer-protospacer match triggers Cas activity to repress the provirus and maintain it in the lysogenic state. The proviruses could not be induced with mitomycin C treatment.

The detection of integrated proviruses in the *Halococcus* genomes reported here, together with the presence of a variety of anti-viral defence mechanisms, indicates that *Halococcus* strains are likely susceptible to viral infection, at least by Caudoviricetes-like viruses. Previously, Xu et al. reported the discovery of a provirus in the *Hcc. hamelinensis* 100A6 genome that shares <5% sequence similarity with known haloarchaeal viruses and may represent a novel family. (Xu et al., 2024). Moreover, the proviruses in *Halococcus* sp. H9 and *Halococcus* sp. H11 also show low similarity (<10%) to known archaeal viruses and to the provirus in *Hcc. hamelinensis* 100A6, suggesting that these proviruses may belong to a novel family within the Caudoviricetes. In contrast, the predicted proviruses share moderate similarity (20–50%) with another *Halococcus* provirus that we predicted from a publicly available genome (Fig. 6). Currently, no free-virus particles infecting *Halococcus* have been described.

## Conclusion

This research expands the genomic and physiological understanding of the *Halococcus* genus by presenting high-quality draft genomes of seven strains and highlighting significant interspecies variation in growth patterns, genome structure, defense mechanisms, and viral interactions. The differences in growth rates among closely related strains, notably the reduced growth rates of those carrying proviruses, suggest that lysogeny has distinct physiological disadvantages, consistent with earlier research in bacteria and other haloarchaea.

Comparative genomic and phylogenetic analyses revealed that traditional marker genes alone are insufficient to resolve *Halococcus* taxonomy, whereas genome-based metrics robustly indicate that most isolates represent novel species candidates. The diverse and uneven distribution of antiviral defense systems further implies that *Halococcus* strains experience distinct ecological and viral pressures in hypersaline environments. Finally, the identification of divergent Caudoviricetes-like proviruses, together with CRISPR spacer-protospacer matches, provides the first genomic evidence for active virus-host interactions in *Halococcus* and suggests the existence of previously unrecognized viral lineages infecting this genus.

## CrediT authorship contribution statement

**Ruby Setiawan:** Writing – review & editing, Writing – original draft, Methodology, Investigation, Data curation, Validation, Visualization, Formal analysis. **Jeroen G. Nijland:** Writing – review & editing, Methodology, Supervision, Validation. **Sabine Schwarzer**: Methodology, Investigation, Visualization. **Thomas Hackl:** Writing – review & editing, Data curation, Validation, Supervision. **Dian Alfian Nurcahyanto:** Writing – review & editing, Investigation. **Ekowati Chasanah:** Writing – review & editing, Resources. **Tessa E.F. Quax:** Writing – review & editing, Supervision, Resources, Funding, Conceptualization, Validation.

## Declaration of competing interest

The authors declare that they have no known competing financial interests or personal relationships that could have appeared to influence the work reported in this paper.

## Data Availability

The assembled genomes of Halococcus strains used in this study were deposited in the ENA database under the accession numbers GCA_980290125, GCA_980293195, GCA_980291705, GCA_980293205, GCA_980293225, GCA_980291625, and GCA_980303575. The raw reads of the WGS project have been deposited in the ENA database under the BioProject accession number PRJEB.106890

## Appendix Supplementary materials

Image, application 1

Image, application 2
