# Supplementary material for: Genome-based taxonomy and provirus identification in Halococcus from hypersaline environments
Source: Curr Res Microb Sci. 2026 Jun 25;11:100637. doi: 10.1016/j.crmicr.2026.100637 (PMC13321033; doi:10.1016/j.crmicr.2026.100637)

Genome-Based Taxonomy and Provirus Identification in *Halococcus* from Hypersaline Environments

Ruby Setiawan^1,3^, Jeroen G. Nijland^1^, Sabine Schwarzer^1^, Thomas Hackl^2^, Dian Alfian Nurcahyanto^3^, Ekowati Chasanah^4^, Tessa E.F. Quax^1^

^1^ Molecular Microbiology, Groningen Biomolecular Sciences and Biotechnology Institute, University of Groningen, 9747AG, Groningen, The Netherlands

^2^ Eco-evolutionary Bioinformatics, Groningen Institute for Evolutionary Life Sciences, University of Groningen, 9747AG, Groningen, The Netherlands

^3^ Research Center for Biosystematics and Evolution, National Research and Innovation Agency (BRIN), 16911, Cibinong, West Java, Indonesia

^4^ Research Center for Applied Microbiology, National Research and Innovation Agency (BRIN), Cibinong, West Java, 16911, Indonesia

**Supplementary Figure S1**. Growth curve of *Halococcus* sp. H9 strain in 20% MGM medium at 37˚C was observed using OD measurements at 600nm over 19 days. Points represent the average values from three biological replicates.


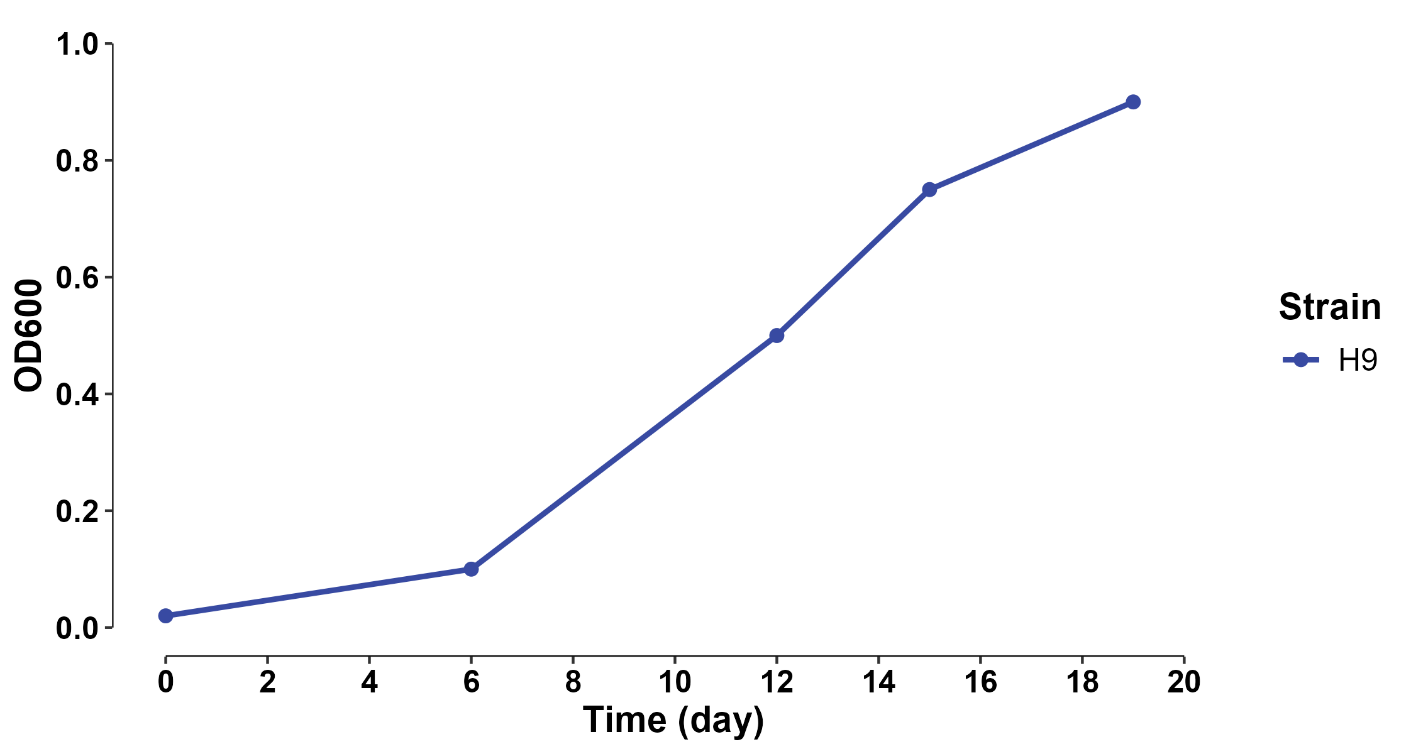


**Supplementary Figure S2.** Heatmap showing copy number variation of KEGG Orthology (KO) genes across *Halococcus* strains. Genes are grouped by functional categories, including central carbon metabolism and nutrient acquisition pathways. The number in the heatmap showed the gene copy number.

**
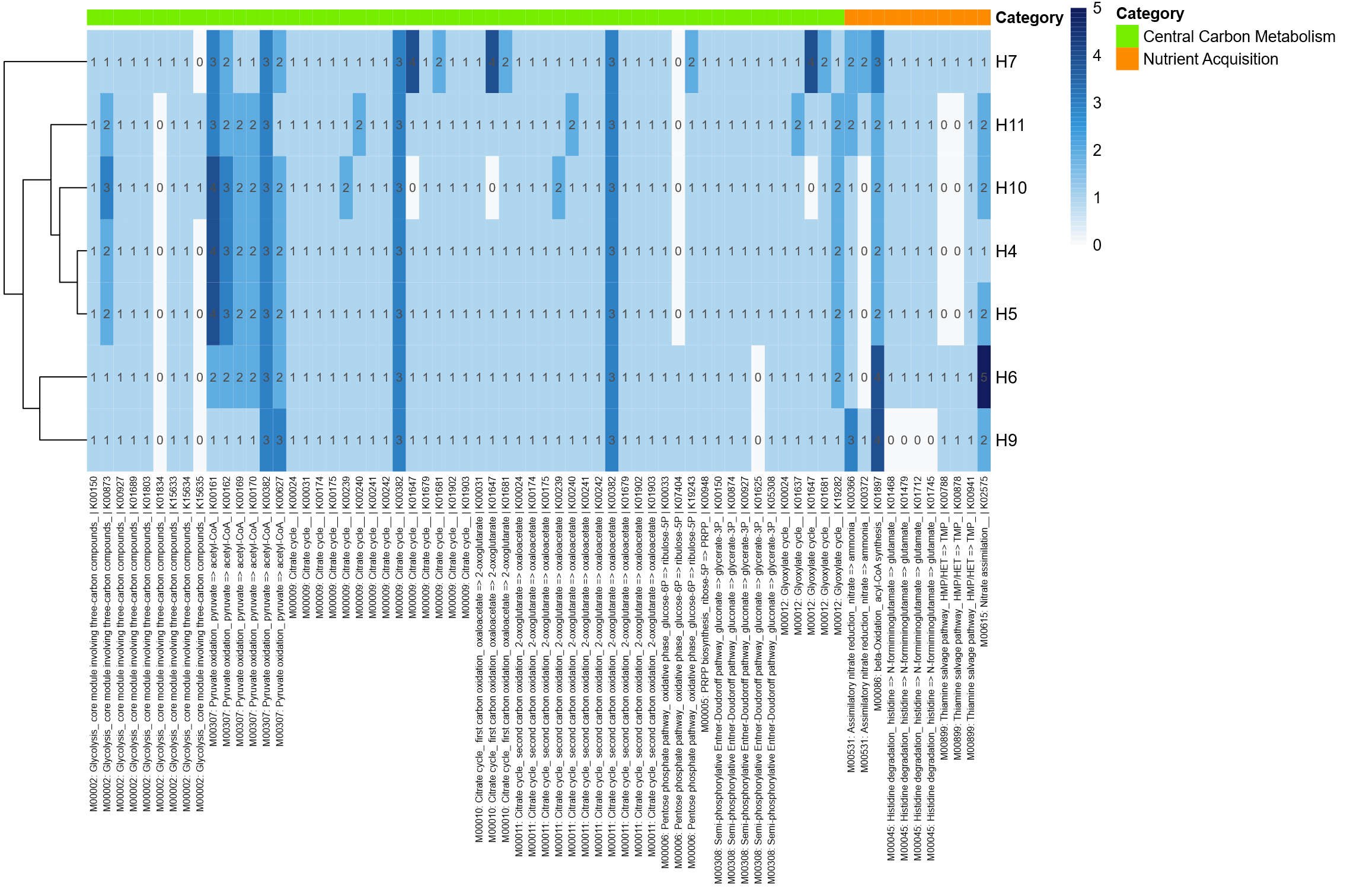
**

**Supplementary Figure S3**. Transmission electron microscopy image of a thin section of *Halococcus* sp. H4 grown in 20% MGM liquid culture. Scale bar: 0.2 µm


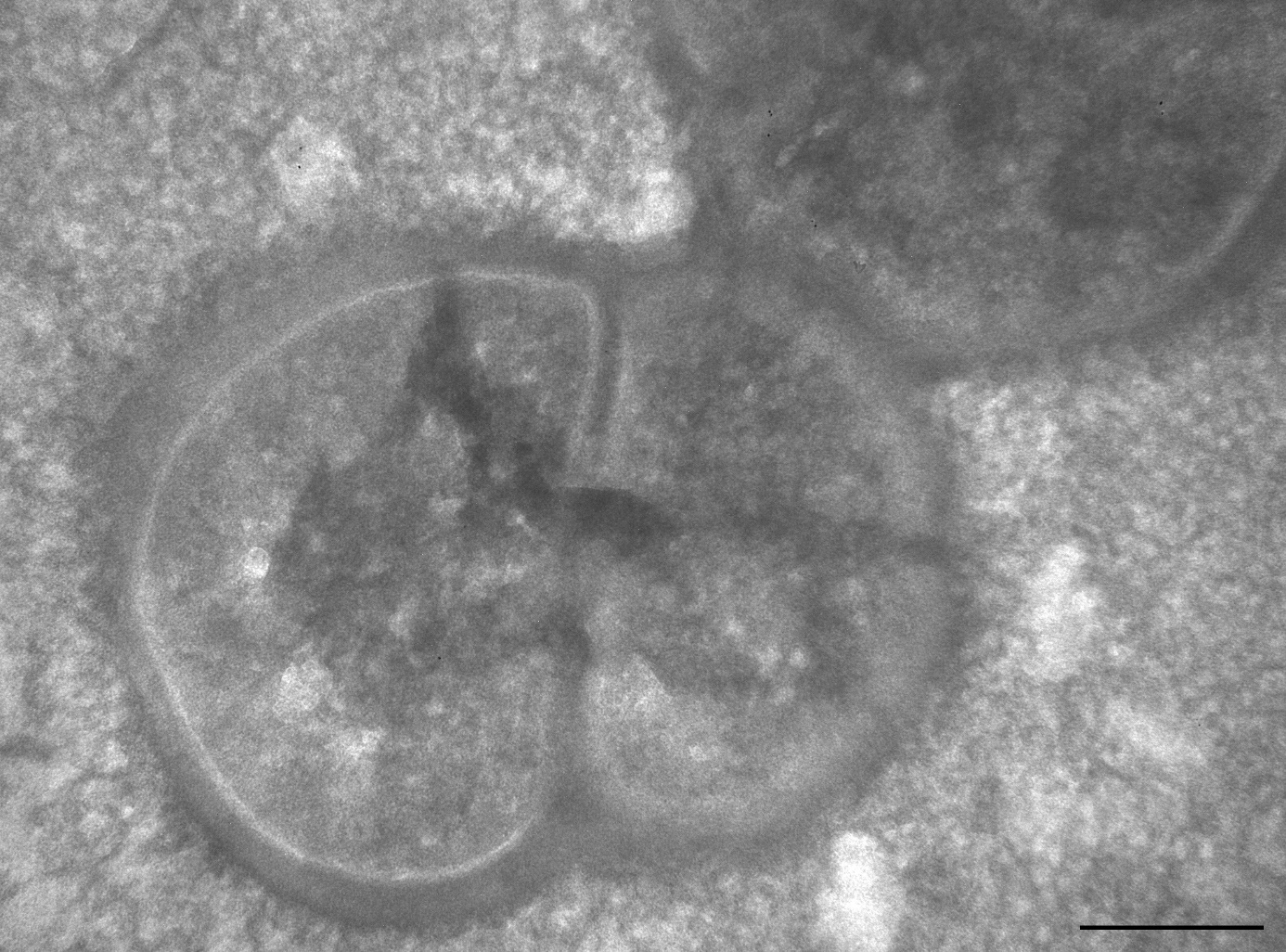


**Supplementary Figure S4.** Scheme of the assembled *Halococcus* genomes. The innermost ring represents the GC skew. The second innermost ring displays the GC content. The third and fourth innermost rings show the positions of tRNA and rRNA, respectively. The outermost red and blue rings are the predicted CDS along the genome from the plus and minus strands.

**
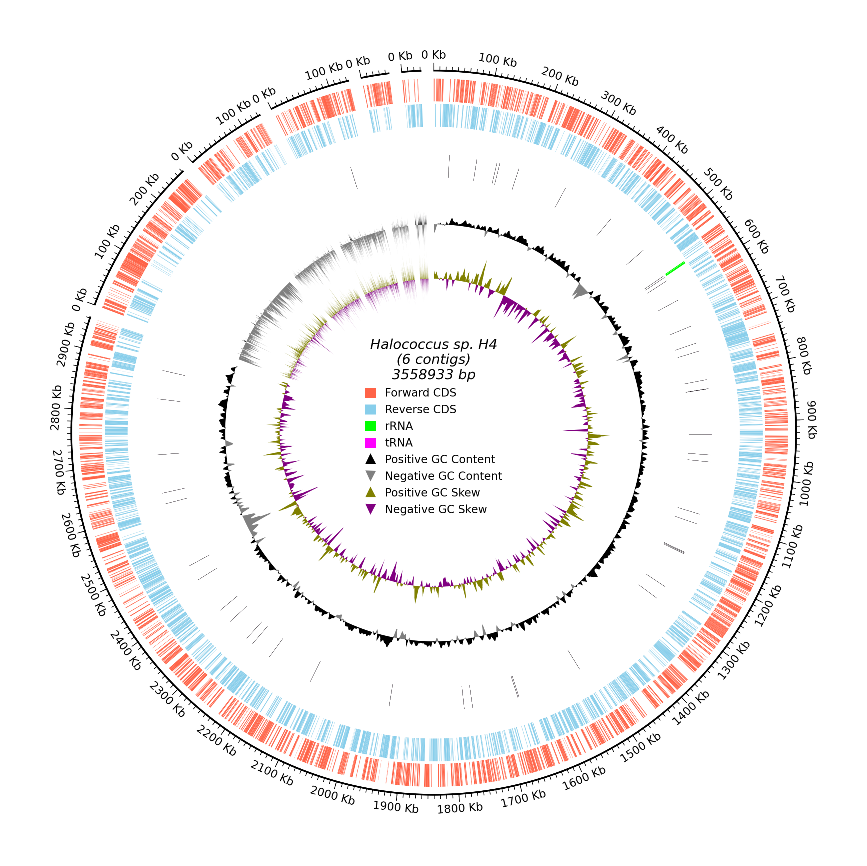

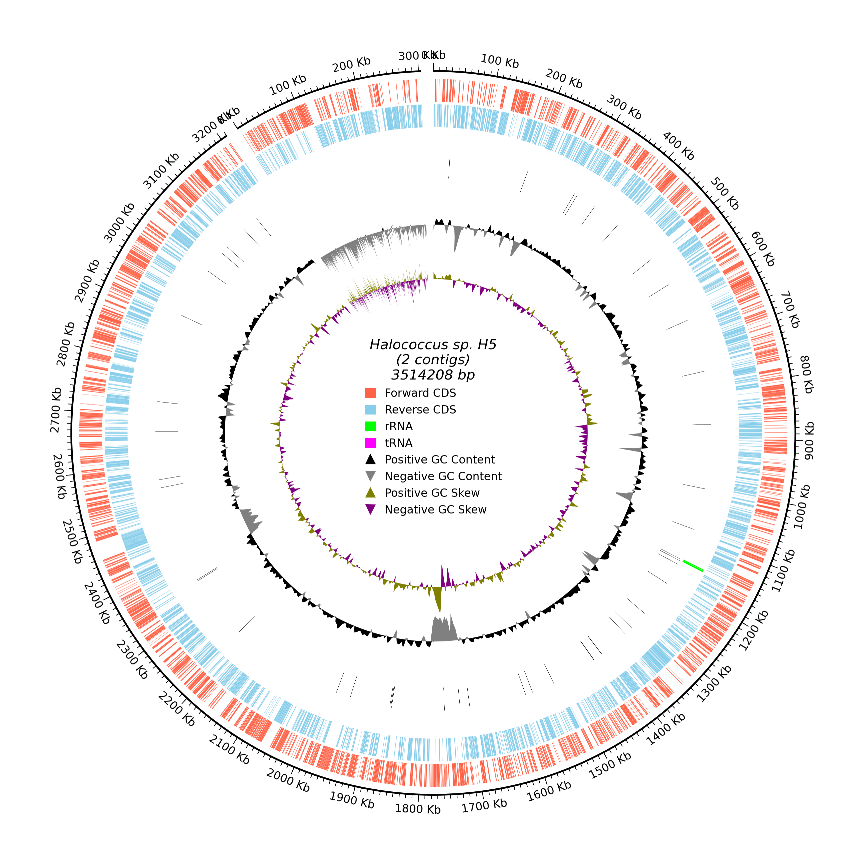

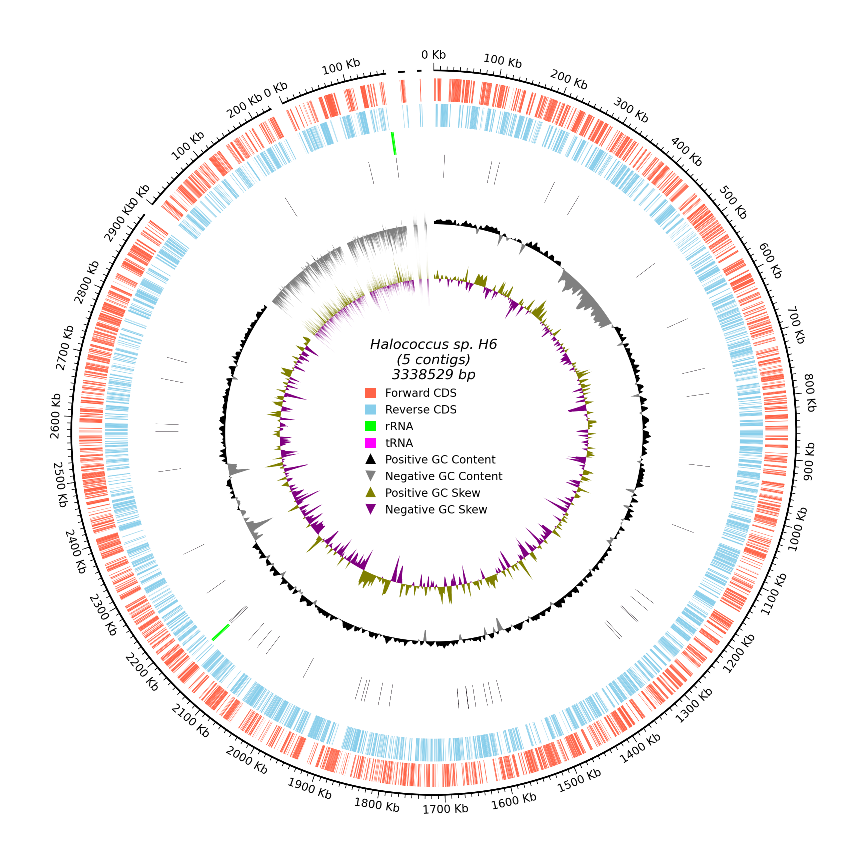

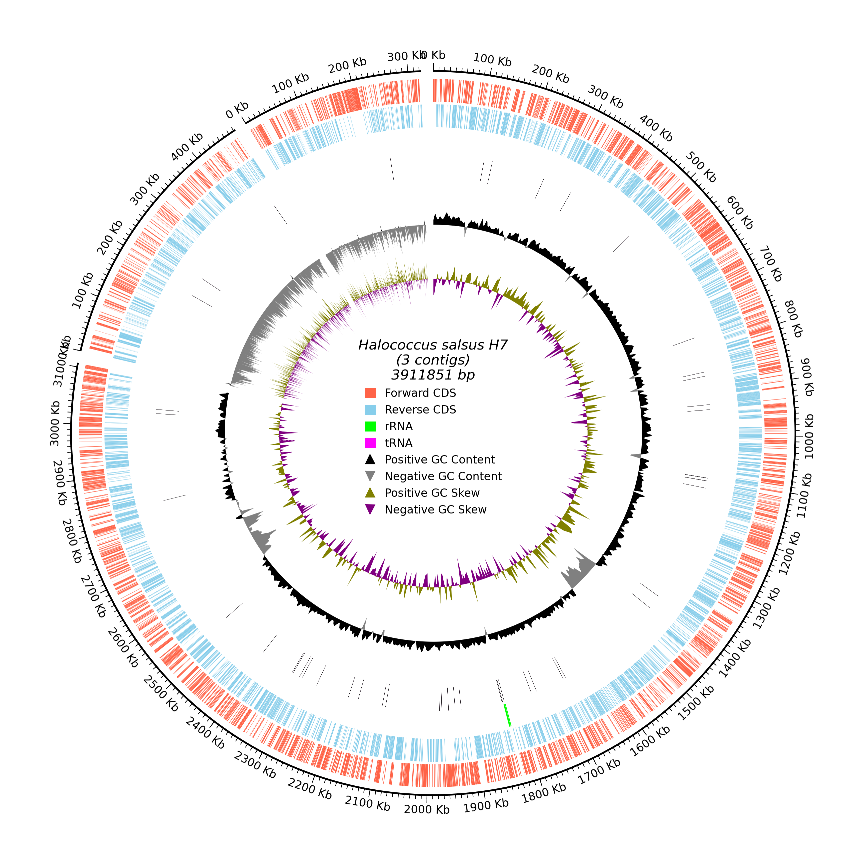

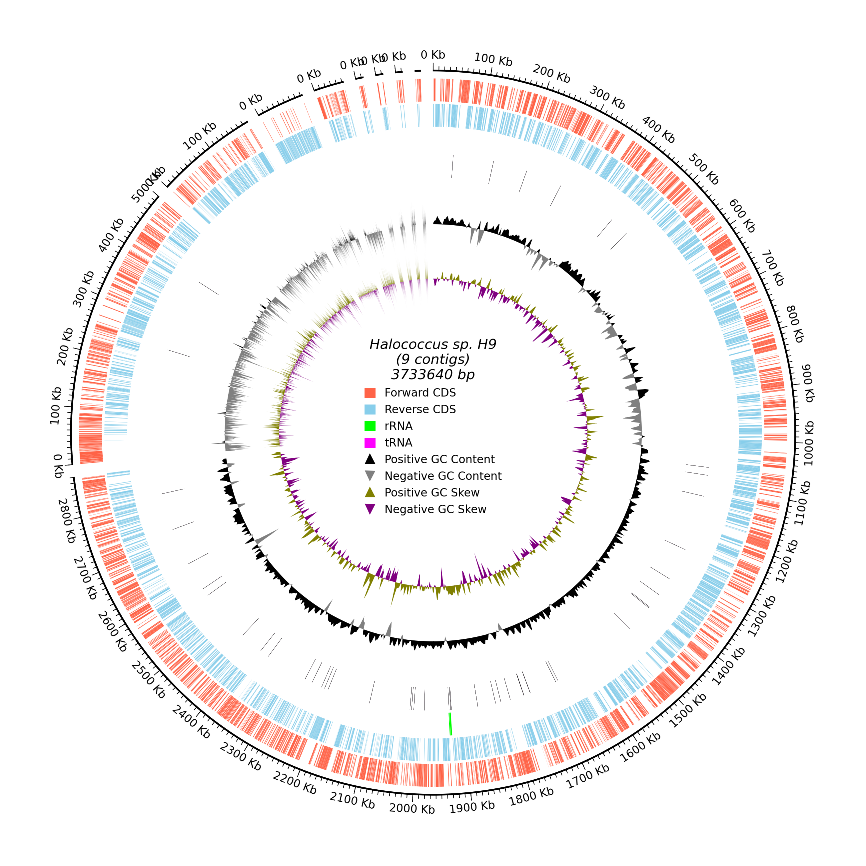

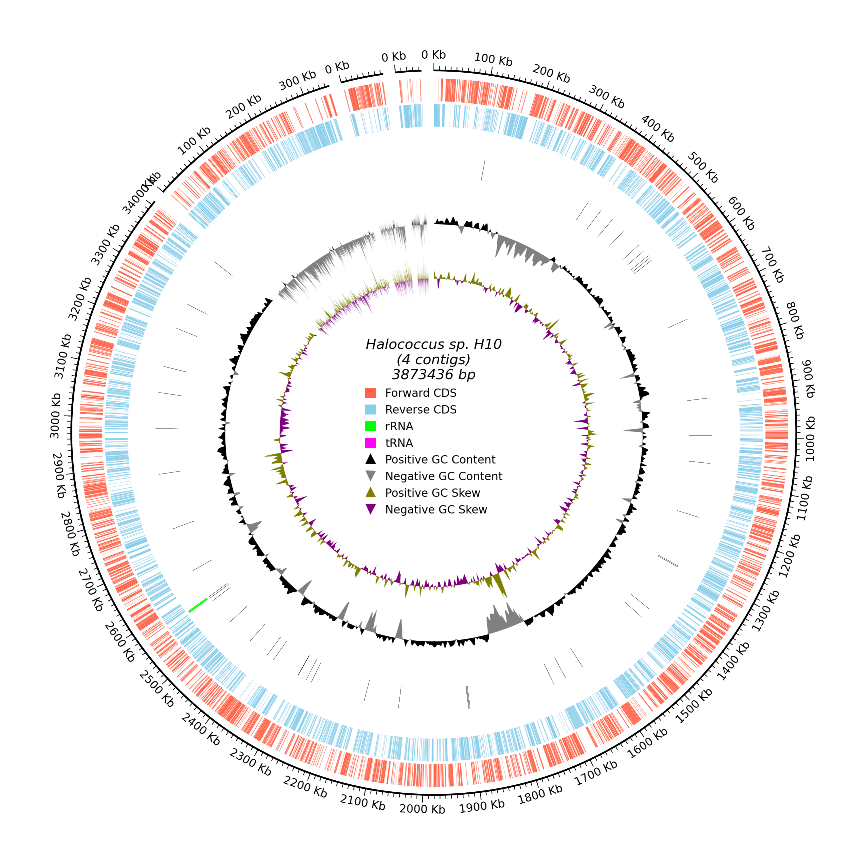

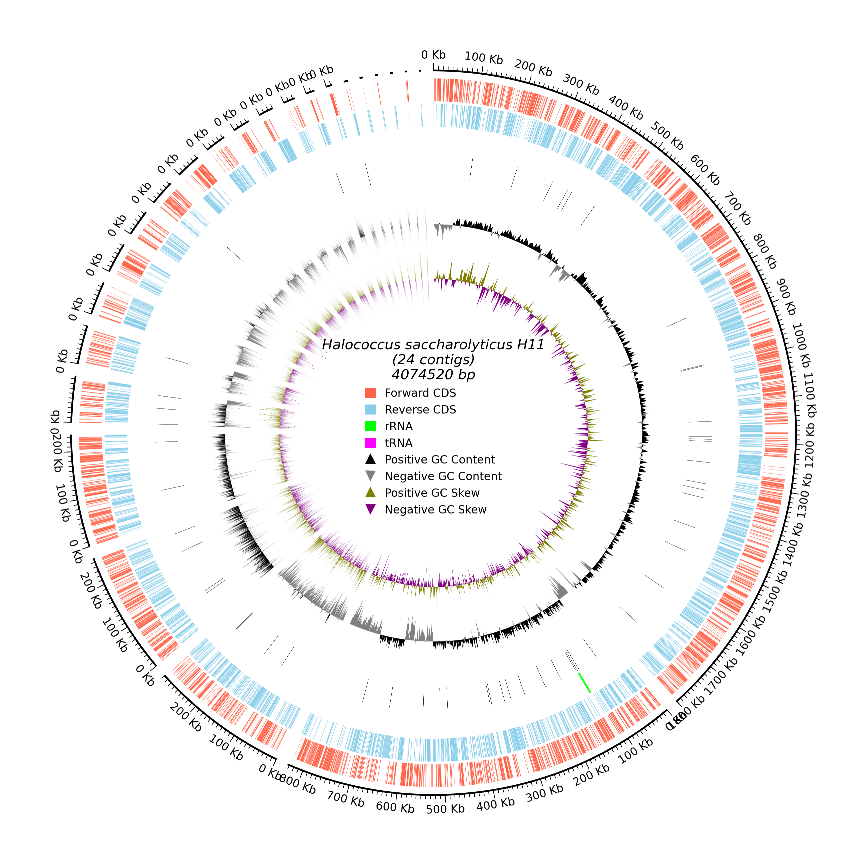
**

**Supplementary Figure S5**.Neighbor-joining phylogenetic tree showing the relationship among *Halococcus* strains analyzed in this study (in bold) and their type strain based on the 16S rRNA gene (A) and the rpoB’ gene (B). The species *Halalkalicoccus tibetensis* JCM 11890T was used as an outgroup. Bootstrap values (BV) < 70%, 70% ≤ BV ≥ 90%, and > 90% (based on 1000 replicates) are shown at branch points as open circles, filled grey circles, and filled black circles, respectively. Sequence accession numbers are shown in parentheses. Bar, 0.05 (A) and 0.01 (B) changes per nucleotide position.

**
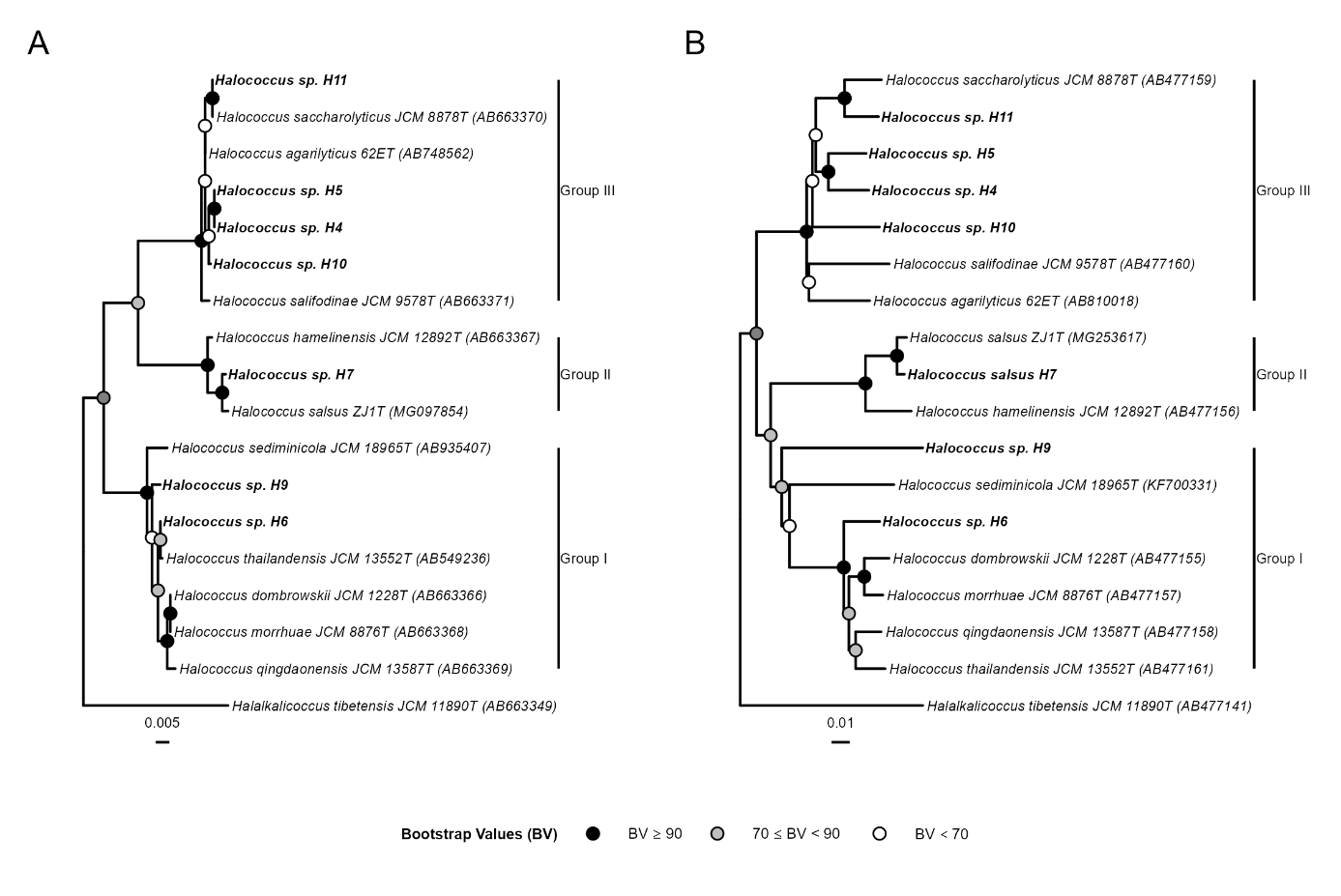
**

**Supplementary Figure S6**. Growth dynamics of *Halococcus* sp. H9 and *Halococcus* sp. H11 under mitomycin C treatments was observed using optical density measurements at 600 nm over 96h. Points represent the average values, and the error bars represent the standard deviation from two biological replicates.


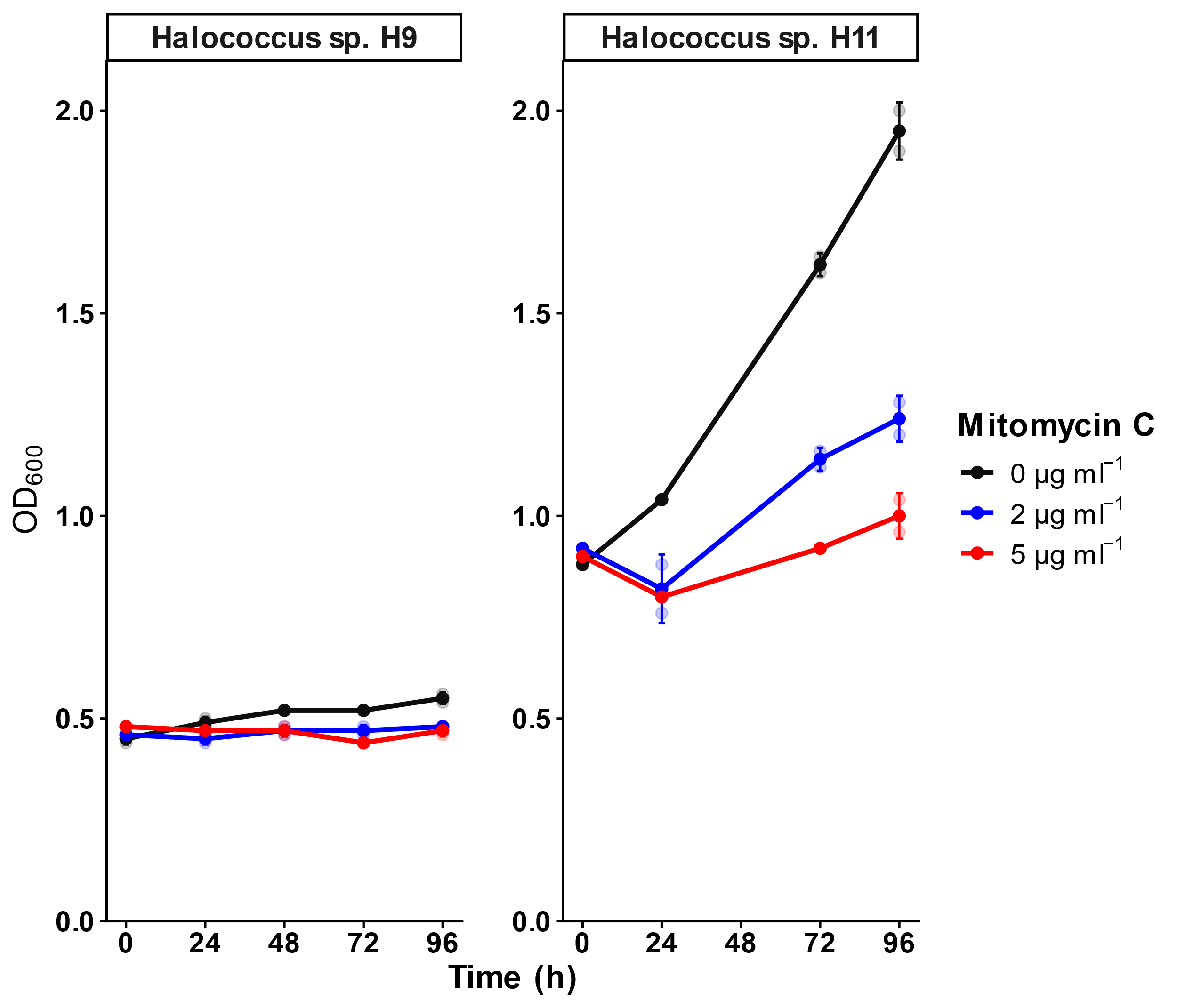


**Supplementary Figure S7**. Relative viral DNA abundance measured by qPCR in *Halococcus* sp. H9 (A) and Halococcus sp. H11 (B) following the mitomycin C treatment. Each strain was analyzed using strain-specific primer sets and independent reference genes for normalization (Supplementary Table S2). The relative expression of viral DNA was calculated using the ΔΔCt method, with controls within each strain used as the reference. Bars indicate mean ± SD. .


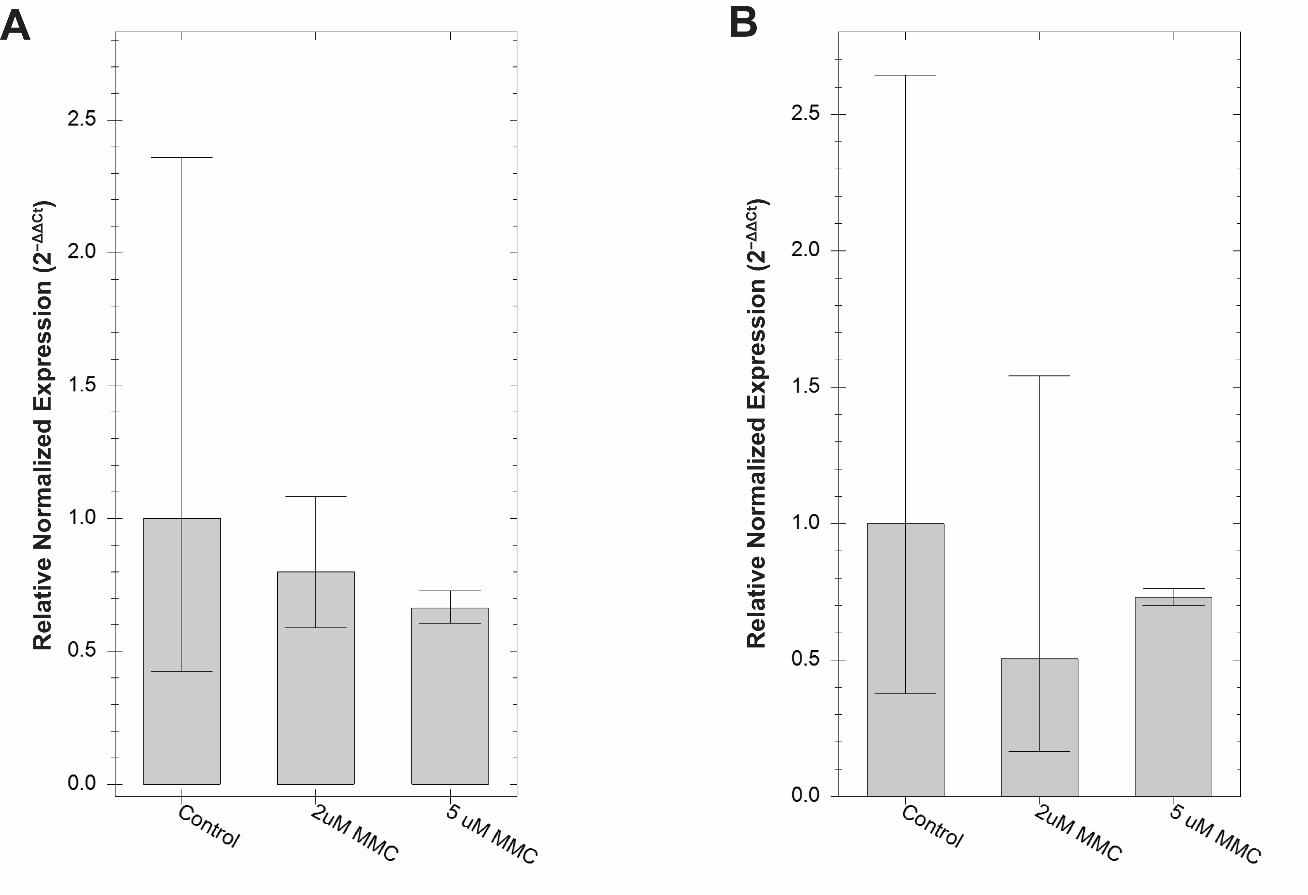

Supplement: Supplementary file 1 [file mmc1.docx]
